# Effect of Previous Exposure to Malaria on Blood Pressure in Kilifi, Kenya: A Mendelian Randomization Study

**DOI:** 10.1161/JAHA.118.011771

**Published:** 2019-03-16

**Authors:** Anthony O. Etyang, Sailoki Kapesa, Emily Odipo, Evasius Bauni, Catherine Kyobutungi, Marwah Abdalla, Paul Muntner, Solomon K. Musani, Alex Macharia, Thomas N. Williams, J. Kennedy Cruickshank, Liam Smeeth, J. Anthony G. Scott

**Affiliations:** ^1^ KEMRI‐Wellcome Trust Research Programme Kilifi Kenya; ^2^ London School of Hygiene and Tropical Medicine London United Kingdom; ^3^ African Population and Health Research Centre Nairobi Kenya; ^4^ Columbia University Medical Center New York NY; ^5^ University of Alabama at Birmingham AL; ^6^ University of Mississippi Medical Center Jackson MS; ^7^ Imperial College London United Kingdom; ^8^ King's College London United Kingdom

**Keywords:** ambulatory blood pressure monitoring, high blood pressure, hypertension, malaria, Mendelian randomization, sickle cell disease, sickle cell trait, thalassemia, High Blood Pressure, Epidemiology, Genetic, Association Studies, Hypertension

## Abstract

**Background:**

Malaria exposure in childhood may contribute to high blood pressure (BP) in adults. We used sickle cell trait (SCT) and α^+^thalassemia, genetic variants conferring partial protection against malaria, as tools to test this hypothesis.

**Methods and Results:**

Study sites were Kilifi, Kenya, which has malaria transmission, and Nairobi, Kenya, and Jackson, Mississippi, where there is no malaria transmission. The primary outcome was 24‐hour systolic BP. Prevalent hypertension, diagnosed using European Society of Hypertension thresholds was a secondary outcome. We performed regression analyses adjusting for age, sex, and estimated glomerular filtration rate. We studied 1127 participants in Kilifi, 516 in Nairobi, and 651 in Jackson. SCT frequency was 21% in Kilifi, 16% in Nairobi, and 9% in Jackson. SCT was associated with −2.4 (95% CI, −4.7 to −0.2) mm Hg lower 24‐hour systolic BP in Kilifi but had no effect in Nairobi/Jackson. The effect of SCT in Kilifi was limited to 30‐ to 59‐year‐old participants, among whom it was associated with −6.1 mm Hg (CI, −10.5 to −1.8) lower 24‐hour systolic BP. In pooled analysis allowing interaction by site, the effect of SCT on 24‐hour systolic BP in Kilifi was −3.5 mm Hg (CI, −6.9 to −0.1), increasing to −5.2 mm Hg (CI, −9.5 to −0.9) when replacing estimated glomerular filtration rate with urine albumin to creatinine ratio as a covariate. In Kilifi, the prevalence ratio for hypertension was 0.86 (CI, 0.76–0.98) for SCT and 0.89 (CI, 0.80–0.99) for α^+^thalassemia.

**Conclusions:**

Lifelong malaria protection is associated with lower BP in Kilifi. Confirmation of this finding at other sites and elucidating the mechanisms involved may yield new preventive and therapeutic targets.

## Abstract 2

See Editorial by Hotez

**Clinical Perspective**

**What Is New?**

- We tested the hypothesis that previous exposure to malaria is associated with increased blood pressure (BP).
- We compared BP in individuals with and without sickle cell trait, which protects against malaria, at 3 sites, 1 where there is ongoing malaria transmission and 2 where there is no malaria transmission.
- We found that individuals with sickle cell trait had lower BP than those without sickle cell trait, but this was only in the area with malaria transmission, indicating that malaria is associated with high BP.

**What Are the Clinical Implications?**

- Confirmation of the existence of a link between previous exposure to malaria and high BP at other sites and elucidation of the mechanisms involved may help in finding new ways to prevent and treat high BP.

## Introduction

High blood pressure (BP) is a major cause of morbidity and mortality worldwide, and its impact is particularly substantial in sub‐Saharan Africa.^1^ Examining factors unique to, or more prevalent in, sub‐Saharan Africa might reveal pathophysiological mechanisms underlying hypertension that could be exploited to reduce the burden of hypertension. More than half of Africa's population lives in areas with moderate to high malaria transmission.^2^ Falciparum malaria is associated with low birth weight, childhood malnutrition, and chronic inflammation, each of which has been associated with the development of hypertension.^3^ In addition, children of women who experienced malaria in pregnancy have higher BP at 1 year of age than those whose mothers did not have malaria during pregnancy.^4^

Mendelian randomization studies, in which genetic polymorphisms are used to represent environmental exposures, are increasingly being applied in determining causality.^5^ These natural experiments, based on genes acquired at conception, overcome the limitations of observational studies while avoiding the expense and ethical concerns that prevent the conduct of randomized clinical trials.

Sickle cell trait (SCT) and α^+^thalassemia are common genetic polymorphisms among African populations that provide protection against malaria. Examining differences in BP among adults with and without these polymorphisms can allow inferences to be made as to whether malaria influences BP.^3^ In the present study, we tested whether SCT and α^+^thalassemia are associated with lower BP and a lower prevalence of hypertension in Kilifi, Kenya, where there is malaria transmission. For comparison, we investigated the same association in Nairobi, Kenya, and Jackson, Mississippi, 2 areas where there is no malaria transmission.

## Methods

### Data and Materials Availability

The authors confirm that, for approved reasons, some access restrictions apply to the data underlying the findings. The authors are unable to make the data more freely available because of the terms for data sharing included in the consent forms for this study. Data are available through the Data Governance Committee of the KEMRI Wellcome Trust Research programme where uses are compatible with the consent obtained from participants for data collection in this study. Requests can be sent to the coordinator of the Data Governance Committee on dgc@kemri-wellcome.org and the Jackson Heart Study on jhspub@umc.edu.

This Mendelian randomization study was performed among residents of 3 sites (Figure S1) with different levels of malaria transmission; Kilifi, Kenya, where there has been moderate malaria transmission,^6^, ^7^, ^8^ and 2 sites (Nairobi, Kenya, and Jackson, Mississippi) where there is no malaria transmission.^9^, ^10^, ^11^ We utilized SCT, which offers 50% to 90% protection against malaria episodes,^12^, ^13^ predominantly in childhood,^14^ to represent malaria exposure (Figure S2). In a secondary analysis among Kenyan participants, we used α^+^thalassemia, which provides a lower level of protection against malaria than SCT. To be eligible for the study in Kenya, individuals had to be 10 years or older at investigation and to be lifelong residents of Kilifi or Nairobi. In Kilifi, we recruited randomly selected residents of Chasimba and Junju locations within the Kilifi Health and Demographic Surveillance System.^15^ Kilifi participants were predominantly of the Chonyi subtribe of the Mijikenda ethnic group. In Nairobi, we recruited randomly selected residents of the Nairobi Urban Health and Demographic Surveillance System.^16^ Study participants in Nairobi were randomly selected from among those who had self‐identified as belonging to ethnic groups known to have a high frequency of malaria‐protective polymorphisms (Luhya, Luo, Teso, Mijikenda).^17^, ^18^ Data were collected in Kenya between December 2015 and June 2017. Participants in the United States were blacks aged 21 years and older recruited into the Jackson Heart Study between 2000 and 2004.^19^

We used appropriately sized cuffs on the nondominant arm to undertake ambulatory BP monitoring (ABPM). We used the Arteriograph24 (TensioMed Ltd.) device in Kenya and the Spacelabs 90207 (Spacelabs) device in the United States. For the primary analyses, we defined completeness of ABPM recordings using European Society of Hypertension (ESH) criteria.^20^ These criteria require ≥20 daytime (9 am–9 pm) and ≥7 nighttime (1 am–6 am) readings.^20^ The respective time periods were used to determine mean daytime and nighttime BP. The mean 24‐hour BP was calculated using all available readings. Prevalent hypertension was diagnosed among participants 16 years and older who met any of the ESH‐defined thresholds for ABPM hypertension regardless of whether they were taking antihypertensive medication. The thresholds used were 24‐hour systolic BP (SBP) ≥130 mm Hg or 24‐hour diastolic BP ≥80 mm Hg, daytime SBP ≥135 mm Hg or daytime diastolic BP ≥85 mm Hg, and/or nighttime SBP ≥120 mm Hg or nighttime diastolic BP ≥70 mm Hg BP.^21^, ^22^ Detailed study procedures are described in the supplement.

### Statistical Analysis

The primary outcome was the difference in 24‐hour SBP by SCT status. We determined that a sample of 1115 participants in Kilifi and 1270 participants in Nairobi and Jackson combined would provide at least 80% statistical power to detect a 4‐mm Hg difference in 24‐hour SBP between participants with and without SCT at each of these sites, assuming an SCT frequency of 15% in Kilifi and 10% in Nairobi/Jackson and an SD for 24‐hour SBP of 15 mm Hg. We defined secondary outcomes as the genotype‐specific differences in systolic and diastolic components of daytime and nighttime BP, and hypertension as defined above. A 2‐sided α level of ≤0.05 was taken to indicate statistical significance.

We used χ^2^ test and Student *t* test to compare categorical and continuous variables at each site by genotype. Hardy–Weinberg equilibrium was evaluated using a χ^2^ test. Nonnormally distributed variables were log‐transformed before analysis.

Two types of analyses were conducted to test the hypothesis. First, we compared BP among participants with and without SCT at each of the 3 sites, while adjusting for confounders as described below. Second, we pooled data from the 3 sites and analyzed whether there was an interaction in the effect of SCT on BP by site.

In the first analyses, which were site‐specific, we performed linear regression to determine whether SCT status was associated with 24‐hour SBP, adjusting for age, sex, and estimated glomerular filtration rate (eGFR)^23^ (Figure S3), which were specified a priori as potential confounders. These covariates were also used in Poisson regression models with robust variance to assess whether SCT was associated with prevalent hypertension. As α^+^thalassemia modifies the protective effect of SCT against malaria,^24^ we tested for statistical interaction in their effect under both dominant and additive conditions among Kilifi participants.

The second, pooled analyses were conducted as follows. Initially, we tested for heterogeneity in the effect of SCT on BP in the 2 sites with no malaria transmission, Nairobi and Jackson, by conducting a linear regression allowing interaction by site. As there was no evidence of heterogeneity in this analysis, we combined data from these 2 sites and then tested whether the effect of SCT on BP differed across sites with different levels of malaria transmission (Kilifi versus Nairobi/Jackson). This was conducted in a regression model that tested for the main effects of age, sex, eGFR, malaria site (Kilifi versus Nairobi/Jackson) and SCT, and an interaction term for SCT and malaria site.

Prespecified subgroup analyses included stratification by age category (10–29, 30–59, and ≥60 years) and sex, and after exclusion of participants taking antihypertensive medication. These subgroup analyses were performed for Kilifi separately and for Nairobi and Jackson combined. We also examined the effect of replacing eGFR with log‐transformed urine albumin to creatinine ratio as the covariate representing renal function in the pooled regression models.

In sensitivity analyses, we repeated the analyses using an expanded data set that included participants whose ABPM data met the less stringent IDACO (International Database of Ambulatory Blood Pressure in relation to Cardiovascular Outcome) study criteria for completeness. These criteria require a minimum of 10 daytime (10 am–8 pm) readings and 5 nighttime (12 am–6 am hours) readings.

All analyses were conducted using Stata version 15 software (StataCorp).

The ethical review committees/institutional review boards of the Kenya Medical Research Institute, London School of Hygiene and Tropical Medicine, Jackson State University, Tougaloo College, and the University of Mississippi Medical Center approved the study. Analysis of Jackson Heart Study data was approved by the University of Alabama at Birmingham's institutional review board. Written informed consent, and assent for participants younger than 18 years in Kenya, was obtained from study participants or parents.

## Results

**Figure 1:**
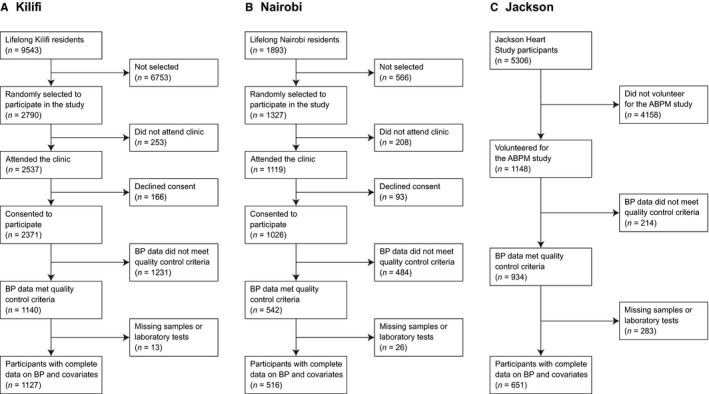
Study flow chart. ABPM indicates ambulatory blood pressure monitoring; BP, blood pressure.

We identified 9543 lifelong residents in the Kilifi population register and, using a random number sequence, we invited 2790 to participate in the study (Figure 1). Characteristics of participants by consent to undergo ABPM and by completeness of ABPM recordings are summarized in Tables S1 and S2, respectively. Individuals with homozygous sickle cell disease (n=5) were excluded from all analyses. Complete data were available for 1127 participants. None of the participants were previously aware of their genetic status. Overall, 238 (21%) participants had SCT and 768 (67%) were either heterozygous (−α/αα) or homozygous (−α/−α) for α^+^thalassemia, equally distributed by SCT status (69% for those with SCT versus 67% for those without SCT, *P*=0.5). There was no departure from Hardy–Weinberg equilibrium for either SCT (*P*=0.5) or α^+^thalassemia (*P*=0.8). Mean 24‐hour SBP was 127±18 mm Hg.

Characteristics of participants by study site and SCT status are presented in Table 1. A higher proportion of participants without SCT versus participants with SCT in Jackson were taking antihypertensive medication. In Kilifi, eGFR was lower among participants with SCT versus participants without SCT. Urine albumin to creatinine ratio was higher among participants with SCT versus participants without SCT in Kilifi and Jackson.

**Table 1:** Characteristics of Study Participants With and Without SCT by Study Site

Characteristic	Kilifi (N=1127)		Nairobi (N=516)		Jackson (N=651)	
	SCT (n=238)	Non‐SCT (n=889)	SCT (n=82)	Non‐SCT (n=434)	SCT (n=58)	Non‐SCT (n=593)
	No. (%)	No. (%)	No. (%)	No. (%)	No. (%)	No. (%)
Women	126 (53)	528 (59)	40 (49)	235 (54)	38 (66)	392 (66)
Smoker	17 (7)	78 (9)	3 (4)	7 (2)	5 (9)	74 (12)
Previously diagnosed with hypertension^a^	37 (16)	127 (14)	7 (9)	53 (12)	31 (53)	367 (62)
Taking antihypertensive medication	9 (4)	26 (3)	0 (0)	8 (2)	26 (46)	338 (61)
	Mean (SD)	Mean (SD)	Mean (SD)	Mean (SD)	Mean (SD)	Mean (SD)
Age, y	41 (22)	39 (22)	20 (14)	23 (18)	61 (12)	60 (11)
BMI, kg/m^2^	20.6 (3.6)	20.6 (3.8)	20.2 (3.9)	20.5 (4.3)	31.1 (7.0)	30.9 (6.3)
HbA_1c_, %	5.2 (0.6)	5.1 (0.8)	5.2 (1.1)	5.2 (1.0)	6.0 (1.2)	6.1 (1.3)
Hemoglobin, g/dL	12.6 (2.0)	12.6 (1.6)	13.4 (1.7)	13.2 (1.7)	12.8 (1.4)	13.0 (1.4)
WBC count ×10^9^/L	5.7 (1.5)	5.7 (1.4)	5.4 (1.4)	5.4 (1.6)	4.9 (1.1)	5.3 (1.5)
Platelet count ×10^9^/L	267 (97)	262 (83)	283 (99)	287 (100)	230 (58)	243 (61)
Plasma osmolality, mOsm/kg	290 (6.6)	290 (5.8)	291 (10)	291 (11)	··· (···)	··· (···)
eGFR, mL/min per 1.73 m^2^	108 (35)	114 (42)	118 (27)	115 (24)	85 (26)	87 (26)
Log UACR, mg/g	1.3 (0.5)	1.2 (0.6)	1.3 (0.7)	1.5 (0.7)	1.06 (0.6)	0.90 (0.5)

Plasma osmolality measurements were not available for Jackson participants. BMI indicates body mass index; eGFR, estimated glomerular filtration rate; HbA_1c_, glycosylated hemoglobin; SCT, sickle cell trait; UACR, urine albumin to creatinine ratio; WBC, white blood cell.

a Answered “yes” to the question: Has a doctor or healthcare worker previously told you that you have high blood pressure?

**Figure 2:**
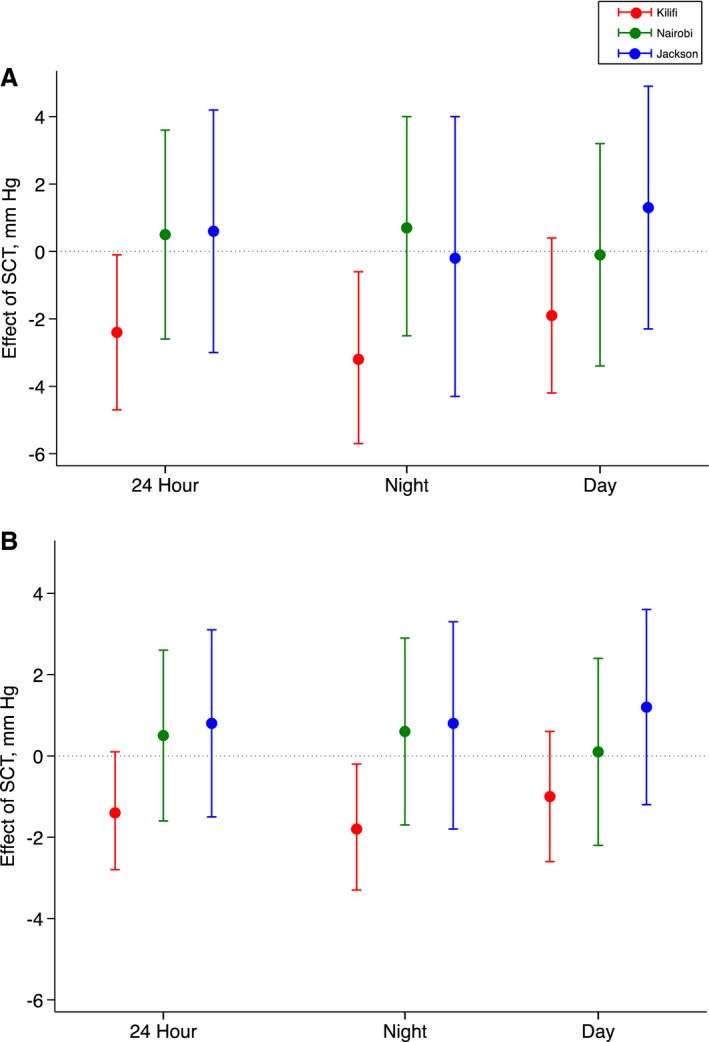
Effect of sickle cell trait (SCT) on blood pressure by study site. **A**, Systolic blood pressure and (**B**) diastolic blood pressure. Linear regression models with adjustment for age, sex, and estimated glomerular filtration rate.

Mean 24‐hour SBP in Kilifi was 126±18 mm Hg in individuals with SCT and 127±18 mm Hg in individuals without SCT. In Nairobi/Jackson, mean 24‐hour SBP was 123±13 mm Hg in individuals with SCT and 123±14 mm Hg in individuals without SCT. After adjusting for age, sex, and eGFR in regression analyses, SCT was associated with a −2.4 (95% CI, −4.7 to −0.2) mm Hg (*P*=0.037) lower 24‐hour SBP in Kilifi (Figure 2). In Nairobi and Jackson, there was no association between SCT and 24‐hour SBP. As there was no heterogeneity between Nairobi and Jackson in the effect of SCT on any BP measure (test for interaction, *P*=0.409–0.944; Table S3) data from these sites were pooled in subsequent analyses. The proportion of variation in BP explained by the regression model as determined by the adjusted *r* ^2^ statistic was 25% in Kilifi, 16% in Nairobi, and 7% in Jackson.

The association between SCT and 24‐hour SBP observed in Kilifi was unaffected by excluding participants taking antihypertensive medication (Table S4).

In Kilifi, SCT was associated with lower 24‐hour SBP (−6.1 mm Hg; 95% CI, −10.5 to −1.8 [*P*=0.006]) among participants aged 30 to 59 years but not in participants in the other age categories (Table 2). In Nairobi and Jackson combined, there was no association between SCT and any BP measure in any age group except for 24‐hour and daytime diastolic BP in those 60 years and older, where BP was higher among participants with SCT. In Kilifi, there was a numerically stronger association between SCT and lower 24‐hour SBP in women compared with men but there was no statistically significant interaction by sex (*P*=0.218, Table S5).

**Table 2:** Age‐Specific Effects of SCT on BP by Study Site

Age, y	No.	24‐h BP				Nighttime BP				Daytime BP			
		SBP	95% CI	DBP	95% CI	SBP	95% CI	DBP	95% CI	SBP	95% CI	DBP	95% CI
Kilifi													
10 to 29	494	−0.1	−3.2 to 1.3	−.05	−1.6 to1.5	−0.8	−3.2 to 1.7	0.05	−1.7 to 1.8	−1.5	−4.0 to 0.9	−0.1	−1.6 to 2.0
30 to 59	384	−6.1	−10.5 to −1.8	−4.5	−7.4 to −1.6	−7.6	−12 to −2.9	−4.9	−7.9 to −2.0	−4.7	−9.1 to −0.3	−3.9	−7.0 to −0.8
≥60	249	0.2	−6.1 to 6.5	1.0	−2.8 to 4.8	−1.3	−8.7 to 6.1	−0.3	−4.4 to 3.9	1.6	−4.6 to 7.8	1.9	−2.1 to 5.8
Nairobi and Jackson pooled together^a^													
10 to 29	399	0.6	−2.3 to 3.5	0.6	−1.2 to 2.5	1.1	−1.8 to 4.0	0.9	−1.2 to 2.8	−0.4	−3.6 to 2.8	−.04	−2.2 to 2.1
30 to 59	389	−1.3	−6.1 to 3.4	−1.3	−4.4 to 1.8	−1.5	−6.8 to 3.9	−0.5	−3.8 to 3.2	−1.0	−5.7 to 3.7	−1.2	−4.5 to 2.1
≥60	378	3.8	−1.4 to 8.9	4.1	0.8–7.3	2.1	−3.8 to 8.1	3.5	−0.2 to 7.0	4.8	−0.5 to 10	4.8	1.4–8.2

Results of linear regression models adjusted for age, sex, and estimated glomerular filtration rate. BP indicates blood pressure; DBP, diastolic blood pressure; SBP, systolic blood pressure; SCT, sickle cell trait.

a Participants in Jackson were 21 years and older.

**Figure 3:**
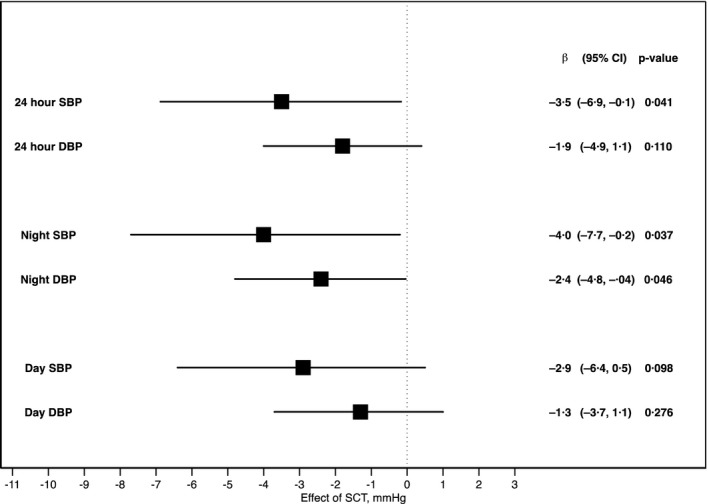
Effect of sickle cell trait on blood pressure (BP) in Kilifi vs Nairobi and Jackson pooled together. Points (and bars) represent the difference in BP (and 95% CI), measured in mm Hg, associated with sickle cell trait (SCT) in Kilifi (a malaria site) vs Nairobi/Jackson (nonmalaria sites). Estimates were derived as the interaction term (malaria vs nonmalaria sites) in a linear regression of BP by SCT status after adjusting for age, sex, and estimated glomerular filtration rate. DBP indicates diastolic blood pressure; SBP, systolic blood pressure.

In the pooled analyses, SCT was associated with −3.5 mm Hg (CI, −6.9 to −0.1) lower 24‐hour SBP (*P*=0.041) in Kilifi when compared with Nairobi and Jackson (Figure 3). This interaction model was associated with an adjusted *r* ^2^ statistic of 20%. The magnitude of the difference was larger (−5.2 mm Hg; CI, −9.5 to −0.9 [*P*=0.019]) when we adjusted for log‐transformed urine albumin to creatinine ratio instead of eGFR (Table S6). Stratified by sex, the effect of SCT on 24‐hour SBP in Kilifi compared with Nairobi/Jackson was not stronger in women compared with men (*P*=0.419, Table S7).

The prevalence of hypertension among study participants was 56% in Kilifi, 28% in Nairobi, and 62% in Jackson. In Kilifi, the adjusted prevalence ratio (PR) for hypertension among participants with SCT versus those without SCT was 0.86 (CI, 0.76–0.98, *P*=0.027). In Nairobi and Jackson, the corresponding adjusted PR was 0.96 (CI, 0.80–1.16; *P*=0.706). When using clinic BP to define hypertension (SBP ≥140 and/or diastolic BP ≥90 mm Hg), the adjusted PR for hypertension among participants with SCT versus those without SCT in Kilifi was 0.82 (CI, 0.64–1.05; *P*=0.113). In Nairobi and Jackson, the corresponding adjusted PR when using clinic BP was 1.45 (CI, 0.62–3.42; *P*=0.396).

α^+^Thalassemia deletions were not associated with the primary outcome in either Kilifi and Nairobi (Table S8), but a significant association with prevalent hypertension was present in Kilifi. In Kilifi, the presence of ≥1 thalassemia deletion was associated with an adjusted PR for hypertension of 0.89 (CI, 0.80–0.99; *P*=0.036). There was no effect of α^+^thalassemia on hypertension in Nairobi (PR, 1.43; CI, 0.95–2.15 [*P*=0.085]).

There was no interaction between α^+^thalassemia and SCT on 24‐hour SBP in Kilifi (test for interaction, *P*=0.865).

### Results Based on IDACO ABPM Criteria

The results of sensitivity analyses are presented in Tables S9 through S15. The number of participants who satisfied IDACO criteria for completeness of ABPM data was 2048 (86%). As expected, the associations between SCT and α^+^thalassemia and BP were weaker than in the primary analyses, but the associations with hypertension remained significant. In Kilifi, SCT was associated with an adjusted PR for hypertension of 0.89 (CI, 0.80–0.99; *P*=0.025). In Nairobi and Jackson, the corresponding adjusted PR was 0.96 (CI, 0.81–1.14; *P*=0.659). The presence of any α^+^thalassemia deletion among individuals in Kilifi was associated with an adjusted PR of hypertension of 0.92 (CI, 0.84–0.99; *P*=0.037). In Nairobi, the corresponding adjusted PR for α^+^thalassemia was 1.32 (CI, 0.95–1.86 [*P*=0.096]).

## Discussion

In this study, SCT, a genetic polymorphism associated with partial protection against malaria, was associated with a 2.4‐mm Hg lower mean 24‐hour SBP and a 14% lower prevalence of hypertension in Kilifi, an area with malaria transmission, but not in Nairobi and Jackson, areas with no malaria transmission. α‐Thalassemia, which provides a lower level of protection against malaria was associated with an 11% reduction in the prevalence of hypertension in Kilifi but not in Nairobi. This suggests that increased risk of malaria is associated with higher adult BP. In the absence of malaria, in Nairobi and Jackson, mean BP estimates were marginally higher among participants with SCT than those without SCT. Incorporating this baseline observation in a pooled analysis that compared malaria with nonmalaria sites, we estimate that malaria is responsible for a mean increase in 24‐hour SBP of 3.5 mm Hg.

This difference in BP is roughly similar to those attributed to reduction of salt intake by ≈4 g/d^25^ or a dose of 10 mg/d of ramipril in the HOPE (Heart Outcomes Prevention Evaluation) trial.^26^ At the population level, a reduction in SBP of 3 mm Hg may avert a substantial number of cardiovascular events including an ≈15% reduction in the incidence of stroke.^27^ However, several factors suggest that the actual effect of malaria on BP might be greater. First, SCT is only associated with a 50% reduction in incidence of nonsevere malaria^13^ and 90% reduction against severe malaria episodes,^28^ and this relative protection wanes with age.^14^ Second, the protection offered by SCT against malaria is reduced in individuals with concurrent α^+^thalassemia^24^ who comprised 67% of participants in the current study who had SCT. The current study was not powered to analyze the effect of this interaction. In addition, because Kilifi has low to moderate malaria transmission,^6^ the effect of malaria on BP in other parts of Africa where malaria is endemic could be considerably higher.

In this epidemiological study, we were not able to study the physiological mechanisms by which malaria results in higher BP. However, there are a number of plausible hypotheses. For example, hypertension may be the consequence of chronic inflammation in childhood induced by malaria^3^ and inflammation, itself, has been associated with the development of hypertension.^29^ Malaria also causes stunting and malnutrition, which could influence BP,^3^ although anthropometric indices such as BMI were similar in the groups we studied. The numerically stronger association that we observed between SCT and BP in women could be explained by SCT‐mediated protection against malaria in pregnancy.^30^ Malaria in pregnancy has been associated with gestational hypertensive disorders that place women at risk for chronic hypertension. CD4^+^ and CD8^+^ T‐cells, which play a role in responses to malaria^31^ as well as partially explain sex differences in hypertension,^32^ could possibly explain the sex differences that we observed. Theoretically, BP could be modulated by exposure to antimalarial treatment, but this seems an unlikely explanation because no commonly used antimalarial drug is known to elevate BP over the long term. Elucidating mechanisms by which malaria leads to higher BP could drive the design of novel therapies for the prevention or treatment of hypertension.

We studied populations in Nairobi and Jackson that had not been exposed to malaria to exclude the possibility that the lower BP associated with SCT in Kilifi could have been caused by factors other than malaria (illustrated in Figure S4). Although the study was not powered to detect small changes in BP at these sites, there was no statistically significant difference in BP by SCT status at these 2 sites and there was no evidence of heterogeneity by site. Other studies also suggest that SCT does not influence BP independently. In a study of 15 975 black patients, where SCT was associated with an increased risk of kidney disease, there was no difference in baseline clinic BP, based on SCT.^23^ There was no difference in incident hypertension among participants with or without SCT in an analysis of 1995 black patients followed for 25 years.^33^ A preliminary analysis from the present data set, restricted to 11‐ to 17‐year‐old Nairobi residents, showed that SCT and α^+^thalassemia do not directly influence BP.^34^, ^35^ In addition, large genome‐wide association studies have not reported a statistically significant association between SCT and BP.^36^, ^37^, ^38^

To invalidate the interpretation of malaria as the cause of elevated BP in this study it would be necessary for SCT and α^+^thalassemia, or loci in linkage disequilibrium with them, to be associated with a large reduction in BP. This is highly unlikely because: (1) none of the loci in the Bantu/Central African Republic haplotype that is predominant among individuals with SCT in Kilifi has been associated with BP traits; (2) studies in the United States show that SCT does not reduce the risk of hypertension‐related outcomes such as stroke, heart failure, and chronic kidney disease^23^, ^39^, ^40^ as would be expected if it lowered BP; and (3) most genetic polymorphisms influencing BP tend to have small effects.^36^, ^37^

### Study Strengths

This study had several strengths. First was the use of ABPM, considered the reference standard for measuring BP.^41^ By performing multiple repeated measurements, ABPM gives more accurate estimates of BP.^20^ The largest differences were observed in the primary analyses that utilized more stringent quality criteria, which provided for better accuracy in measurement without introducing a selection bias. In addition, in both the primary and sensitivity analyses, the largest differences were observed when comparing nighttime measurements, which are less susceptible to interference by daytime activities. Nocturnal BP indices are also more predictive of cardiovascular outcomes than daytime or 24‐hour values.^20^, ^42^, ^43^ Second, the Mendelian randomization approach that we used is a robust design for elucidating causal relationships.^5^ We used genetic variants that are strongly associated with malaria and showed associations with the outcomes that were consistent with the different levels of protection against malaria afforded by each variant. Third, study participants in Kilifi were of the same ethnicity, minimizing the possibility that population stratification could explain the differences observed. Fourth, we used prospectively collected health and demographic surveillance system records^15^, ^16^ to ascertain residence in malaria/nonmalaria sites.

### Study Limitations

Although Mendelian randomization is a well‐established method for inferring causality, there are some residual limitations. As we did not have medical record data for the participants in Kilifi, we could not determine the timing, number, or severity of malaria episodes required to elevate BP in adult life. These questions could be investigated using sequential birth cohort studies that take into account the fact that there has been a marked reduction in malaria transmission in Kilifi from the year 2000.^7^ In addition, replication studies in other areas with malaria transmission are needed to confirm the observations we made in Kilifi.

## Conclusions

SCT was associated with lower BP and reduced prevalence of hypertension in Kilifi but not in Nairobi, Kenya, or Jackson, Mississippi, an observation compatible with a causal association between malaria and higher BP. One implication is that malaria elimination would lead to health benefits well beyond those currently described. A second implication is that elucidating the mechanisms by which malaria leads to an elevation in BP could yield new preventive strategies for hypertension and consequent cardiovascular disease.

## Sources of Funding

Etyang, Smeeth, Williams, and Scott are funded by the Wellcome Trust (Fellowship numbers: 103951, 098532, 091758 and 202800, and 098504). Muntner was supported by grant 2R01 HL117323 from the National Heart, Lung, and Blood Institute (NHLBI) and grant 15SFRN2390002 from the American Heart Association. The Jackson Heart Study is supported and conducted in collaboration with Jackson State University (HHSN268201300049C and HHSN268201300050C), Tougaloo College (HHSN268201300048C), and the University of Mississippi Medical Center (HHSN268201300046C and HHSN268201300047C) contracts from the NHLBI and the National Institute for Minority Health and Health Disparities. The views expressed in this article are those of the authors and do not necessarily represent the views of the NHLBI; the National Institutes of Health; or the US Department of Health and Human Services. The funder played no role in the preparation of this article.

## Disclosures

None.

## Supporting information


**Data S1.** Study procedures.
**Table S1.** Characteristics of Those Who Consented to Undergo ABPM vs Those Who Declined
**Table S2.** Characteristics of Participants With and Without Good Quality ABPM Data
**Table S3.** Effect of SCT on BP in Nairobi and Jackson
**Table S4.** Effects of SCT on BP: Effect of Excluding Participants Taking Antihypertensive Medication
**Table S5.** Effect of SCT on BP by Sex and Study Site
**Table S6.** Results of Interaction Analysis Comparing Effect of SCT in Kilifi vs Nairobi/Jackson Using Log Urine Albumin to Creatinine Ratio as a Covariate Instead of Estimated Glomerular Filtration Rate
**Table S7.** Results of Pooled Analysis Comparing the Effects of SCT in Kilifi vs Nairobi/Jackson Stratified by Sex
**Table S8.** Effect of α^+^thalassemia on Ambulatory BP by Study Site
**Table S9.** Characteristics of Study Participants With and Without SCT by Study Site (IDACO Criteria)
**Table S10.** Effect of SCT on BP by Malaria Site (IDACO Criteria)
**Table S11.** Age‐Specific Effects of SCT on BP by Study Site (IDACO Criteria)
**Table S12.** Effect of SCT on BP by Sex and Study Site (IDACO Criteria)
**Table S13.** Effects of SCT on BP: Effect of Excluding Participants Taking Antihypertensive Medication (IDACO Criteria)
**Table S14.** Results of Interaction Analysis Comparing Effect of SCT in Kilifi vs Nairobi/Jackson (IDACO Criteria)
**Table S15.** Effect of α^+^thalassemia on Ambulatory BP by Study Site (IDACO Criteria)
**Figure S1.** Study locations.
**Figure S2.** Causal diagram for the malaria‐high BP hypothesis.
**Figure S3.** Illustrating confounding effect of kidney function (estimated glomerular filtration rate [eGFR]) in individuals with sickle cell trait (SCT).
**Figure S4.** Illustrating confounding caused by pleiotropy.
