# Supplementary material for: Effect of Previous Exposure to Malaria on Blood Pressure in Kilifi, Kenya: A Mendelian Randomization Study
Source: J Am Heart Assoc. 2019 Mar 16;8(6):e011771. doi: 10.1161/JAHA.118.011771 (PMC6475058; doi:10.1161/JAHA.118.011771)
Supplement: Supplementary file 1 — Data S1. Study procedures. Table S1. Characteristics of Those Who Consented to Undergo ABPM vs Those Who Declined Table S2. Characteristics of Participants With and Without Good Quality ABPM Data Table S3. Effect of SCT on BP in Nairobi and Jackson Table S4. Effects of SCT on BP: Effect of Excluding Participants Taking Antihypertensive Medication Table S5. Effect of SCT on BP by Sex and Study Site Table S6. Results of Interaction Analysis Comparing Effect of SCT in Kilifi vs Nairobi/Jackson Using Log Urine Albumin to Creatinine Ratio as a Covariate Instead of Estimated Glomerular Filtration Rate Table S7. Results of Pooled Analysis Comparing the Effects of SCT in Kilifi vs Nairobi/Jackson Stratified by Sex Table S8. Effect of α+thalassemia on Ambulatory BP by Study Site Table S9. Characteristics of Study Participants With and Without SCT by Study Site (IDACO Criteria) Table S10. Effect of SCT on BP by Malaria Site (IDACO Criteria) Table S11. Age‐Specific Effects of SCT on BP by Study Site (IDACO Criteria) Table S12. Effect of SCT on BP by Sex and Study Site (IDACO Criteria) Table S13. Effects of SCT on BP: Effect of Excluding Participants Taking Antihypertensive Medication (IDACO Criteria) Table S14. Results of Interaction Analysis Comparing Effect of SCT in Kilifi vs Nairobi/Jackson (IDACO Criteria) Table S15. Effect of α+thalassemia on Ambulatory BP by Study Site (IDACO Criteria) Figure S1. Study locations. Figure S2. Causal diagram for the malaria‐high BP hypothesis. Figure S3. Illustrating confounding effect of kidney function (estimated glomerular filtration rate [eGFR]) in individuals with sickle cell trait (SCT). Figure S4. Illustrating confounding caused by pleiotropy. [file JAH3-8-e011771-s001.pdf]

Supplemental Material

Data S1.

Study Procedures

a) Study Procedures in Kilifi and Nairobi, Kenya

Participants were recruited from December 2015 to June 2017. We recruited participants aged ≥10 years that were lifelong residents of the Nairobi Urban Health and Demographic Surveillance System[1] and the Kilifi Health and Demographic Surveillance System[2] respectively (locations shown in Figure S1). Lifelong residency was confirmed using prospectively collected residency data from enumeration rounds that are conducted every 3-4 months within the demographic surveillance systems. Continuous residency was required in order to minimize misclassification of exposure to malaria as Nairobi and Kilifi have markedly contrasting malaria transmission patterns. Study participants in Nairobi were randomly selected from those who had self-identified as belonging to ethnic groups known to have a high frequency of malaria protective polymorphisms (Luhya, Luo, Teso, Mijikenda) as a result of hailing from parts of Kenya that are known to be endemic for malaria.[3, 4] Study participants in Kilifi were predominantly from the Chonyi subtribe of the Mijikenda community. The prevalence of hypertension within the Kilifi Health and Demographic Surveillance System which covers an area of 900km^2 is ~17%.[5] However there are significant differences in the incidence to death due to stroke within the study area. Chasimba where >75% of study participants came from has an incidence of death due to stroke that is three times that of Kilifi township, suggesting that there are local geographical differences in the prevalence of hypertension which is the main risk factor for stroke.

In both Kilifi and Nairobi, trained study staff visited all individuals who had been selected to participate in the study at their homes and requested them to come to the

study clinic to undergo study procedures. Those who failed to come to the clinic within 3 months of being requested to do so were considered to have declined our invitation to participate in the study.

At the clinic participants first underwent an interview where they answered questions about their past medical history and their socioeconomic status based on the multi-dimensional poverty (MDP) index.[6] Weight and height were measured using a validated SECA 874™ weighing machine and a portable stadiometer (Seca 213™), respectively. Body mass index was calculated as the weight in kilograms divided by height in meters squared (kg/m^2). We did not classify BMI by age-category in the adolescents that we studied. Mid-upper arm circumference (MUAC) was measured in a standardized manner using TALC™ MUAC tapes. All participants were subsequently fitted with a validated Arteriograph24™(TensioMed Ltd., Budapest, Hungary) device for 24-hour ABPM measurement.[7] The devices were attached on the non-dominant arm and were programmed to take measurements every 20 minutes during daytime hours (0600-2200 hrs) and every 40 minutes at night (2200-0600 hrs). At the end of the 24-hour period, participants returned to the study clinic where the Arteriograph was removed and data downloaded onto computers that would later (within 12 hours) synchronize their data onto an MySQL database hosted on servers located at the KEMRI-Wellcome Trust Research Programme offices in Kilifi, Kenya.

We collected 10ml of blood from participants for full blood count, determination of sickle hemoglobin status and serum electrolytes. After performing automated full blood counts using an ACT 5™ machine, whole blood samples were frozen at -80ºC and then transported to the KEMRI-Wellcome Trust Research Programme laboratories in Kilifi, Kenya for determination of sickle hemoglobin status. DNA was

extracted retrospectively from the frozen samples by use of Qiagen™ DNA blood mini-kits (Qiagen, Crawley, United Kingdom) and typed for sickle hemoglobin and α^{+}thalassemia using polymerase chain reaction. Glycosylated hemoglobin levels were determined using the Biorad™ D-10 machine (Bio-rad Laboratories Inc, Hercules, California).

Serum and urine samples collected from participants were frozen at -80ºC within 4 hours of collection and later transported to the laboratories in Kilifi for analysis. We determined urea and creatinine levels in these samples using ion electrophoresis and the jaffe method, respectively.[8] Creatinine measurements were performed using Isotope dilution mass spectrometry traceable methods. In addition, we determined albumin levels in the urine samples by immunoturbidometry using a Quantex™ microalbumin kit. Estimated glomerular filtration rate (eGFR) was calculated using the Chronic Kidney Disease Epidemiology Collaboration (CKD-EPI) equation in adults and the Schwartz equation in those aged ≤16 years.[9, 10]

b) Study Procedures in Jackson, Mississippi, USA

The Jackson Heart Study (JHS)[11] is a population-based prospective cohort study, which was designed to evaluate cardiovascular disease risk among blacks. The JHS enrolled 5306 noninstitutionalized blacks, aged ≥21 years, between 2000 and 2004. The participants were recruited from the Atherosclerosis Risk in the Community site in Jackson, MS, and a representative sample of urban and rural Jackson, MS, metropolitan tricounty (Hinds, Madison, and Rankin counties) residents, volunteers, randomly selected individuals, and secondary family members.[12] The current analysis was restricted to JHS participants who completed ABPM soon after the baseline study visit (visit 1).

During in-home interviews, trained African American interviewers administered standardized questionnaires to collect self-reported information on socio-demographics (e.g. age, sex, education, marital status and socioeconomic status), previously diagnosed co-morbid conditions and selected health-related behaviors (e.g. current smoking). Weight and height were measured during a clinic visit.

At the clinic visit, blood samples were collected for full blood count, genetic studies and determination of serum sodium, potassium and creatinine concentrations. 24-hour urine samples were collected for determination of creatinine and albumin concentrations. Full blood counts were performed using the Coulter GenS machine (BeckmanCoulter, Hialeah, Florida, USA). DNA was extracted from whole blood samples using Puregene reagents (Gentra System, Minneapolis, USA) and genetic studies were performed as previously described.[12] Biochemical tests were performed using a Vitros 950 or 250 Ortho-Clinical Diagnostics analyzer (Raritan, new Jersey, USA). Urine albumin was measured on a Dade-Behring BN 11 nephelometer (dade-Behring, Newark, Delaware, USA). All tests were performed at the University of Minnesota laboratory with the exception of hematology tests, which were done at the University of Mississippi Medical Center.[13] Creatinine measurements were performed using Isotope dilution mass spectrometry traceable methods.

Participants were fitted with an ABPM device (Spacelabs 90207, Spacelabs, Redmond, WA) on their non-dominant arm. Ambulatory BP was recorded every 20 minutes. After 24 hours, the device was removed, and data were downloaded onto a computer and processed with Medifacts International’s Medicom software (Rockville, MD)

Statistical Methods and Considerations

a) Reporting format

While there are no specific guidelines for reporting Mendelian randomization (MR) studies, the principles outlined in the Strengthening the Reporting of OBsErvational studies (STROBE)[14] guidelines as well as the STROBE Extension for Genetic Association studies (STREGA)[15] were used. Reporting was also guided by the review by Boef *et al*. of the quality of reporting of MR studies.[16]

b) Sample size estimation

The sample size calculation for Kilifi was based on a two-sample t-test comparing mean 24-hour systolic blood pressure in those with and without the sickle cell trait (SCT). The following assumptions were made:

- That the prevalence of SCT would be ≥15%[17]
- That the standard deviation of 24-hour systolic BP would be ≤15 mm Hg[5, 18]

Based on these assumptions we calculated that, for Kilifi, we would need a minimum of 1115 participants with complete data in order to detect a statistically significant 4 mm Hg difference in 24-hour systolic BP with at least 80% statistical power.

For participants in Nairobi/Jackson we assumed that the combined SCT prevalence for the two sites would be ≥ 10%.[19] Other assumptions were similar to those for Kilifi. Based on these assumptions we calculated that for Nairobi/Jackson, we would need minimum of 1270 participants with complete data in order to detect a statistically significant 4 mm Hg difference in 24-hour systolic BP with at least 80% statistical power.

We assumed that with these numbers, we would achieve enough power for the primary outcome measure, a linear regression to determine the effect of SCT on 24-hour BP measures, while adjusting for age, sex and estimated glomerular filtration

rate (eGFR). The literature suggests that the major consideration for sample size calculations in linear regression models is to ensure that there are at least 2-50 individuals per variable in the model[20], a requirement that would almost certainly be achieved if most of the assumptions stated above held true.

c) Quality control criteria for ABPM data

There are 2 internationally recognized quality control criteria used for ABPM data, which are based on completeness of observations. The International Database of Ambulatory blood pressure in relation to Cardiovascular Outcomes (IDACO) study[21] defined ABPM data as acceptable if they include ≥ 10 daytime and ≥ 5 nighttime readings, where daytime is defined as 1000-2000 hrs and nighttime as 0000-0600 hrs.[21] The guidelines from the European Society of Hypertension (ESH) are more stringent; they require ≥20 daytime and ≥7 nighttime readings where daytime is defined as 0900 to 2100 hrs and nighttime as 0100 to 0600 hours.[22] It is important to note that these criteria were arbitrarily set by experts and were not based on outcome studies. As the ESH criteria are more stringent they are likely to lead to a greater loss of data and subsequent loss of power and precision. However, in order to reduce measurement bias and obtain as accurate an effect size as possible, an *a priori* decision was made to restrict our primary analysis to data that met the ESH criteria.

d) Primary and secondary outcome measures

The primary outcome measure was estimated using a linear regression model to determine the effect of SCT on 24-hour systolic blood pressure, after adjusting for age, sex and estimated glomerular filtration rate (eGFR). Blood pressures were obtained by ambulatory blood pressure monitoring using the Arteriograph24™

device.[7] Numerous studies have shown that the more accurate measurements resulting from repeated inflations and more standardized procedures in ABPM make it a much better predictor of cardiovascular events than other BP measurement methods.[22] The justification for adjusting for age, sex and eGFR is given in the section below on confounders and model building.

Secondary outcome measures were defined as follows:

a) effect of α^{+}thalassaemia on 24-hour, daytime and nighttime systolic blood pressures, after adjusting for age, sex and estimated glomerular filtration rate
b) effect of SCT on 24-hour, daytime and nighttime diastolic blood pressures, after adjusting for age, sex and estimated glomerular filtration rate
c) effect of α^{+}thalassaemia on 24-hour, daytime and nighttime diastolic blood pressures, after adjusting for age, sex and estimated glomerular filtration rate
d) prevalence ratio for hypertension in those with and without SCT using log-binomial regression, adjusting for age, sex and estimated glomerular filtration rate

Hypertension was diagnosed by any one of the following criteria in individuals aged ≥16 years: [22, 23]

i) 24-hours systolic BP ≥130 mmHg and/or 24-hour diastolic BP ≥ 90 mm Hg
ii) Daytime systolic BP ≥135 mm Hg and/or daytime diastolic BP ≥ 85 mm Hg
iii) Nighttime systolic BP ≥ 120 mm Hg and/or nighttime diastolic BP ≥ 70 mm Hg

Adjustment for multiple testing was not considered necessary in this scenario of a limited number of clinically relevant pre-specified tests (e.g. compared to GWAS studies)[24]

e) Adjusting for confounders and model building

The theoretical basis for the malaria-high blood pressure hypothesis has been published previously.[25] Briefly, the primary hypothesis is that individuals in Kilifi who were exposed to more malaria disease in childhood (represented by those having haemoglobin AA) would have higher 24-hour systolic blood pressure than those who were exposed to less malaria disease (represented by those having haemoglobin AS [SCT]). The proposed causal diagram drawn purely for purposes of informing the analytical plan can be found in Figure S2.

For purposes of this analysis it is important to note that because malnutrition and stunting are on the causal pathway from malaria to the outcome, adjustment for body mass index (BMI) and other anthropometric indices (e.g. mid upper arm circumference) would be inappropriate.

i) Confounders

The principle of Mendelian randomization holds that because comparisons are based on genetic traits acquired at conception, any relationships between the genetic trait and the outcome are unlikely to be confounded by other exposures as these will be randomly distributed between carriers and non-carriers of the trait.[26] However age, sex, and BMI are known to have a very strong influence on BP and other cardiovascular diseases[27], and are usually adjusted for as 'fixed covariates' in MR/Genome wide association studies [28-31]. We have outlined above why it would be inappropriate to adjust for BMI.

Sickle cell trait has been associated with impaired kidney function as measured by decline in estimated glomerular filtration rate (eGFR) and albuminuria.[19] This association is independent of blood pressure elevation. Impaired kidney function is associated with elevations in blood pressure as a result of sodium retention[32], increased activity of the renin-angiotensin system[33], increased sympathetic activity[34], secondary hyperparathyroidism[35], impaired nitric oxide synthesis[36] and increased prevalence of nocturnal non-dipping BP pattern.[37] It is also possible that kidney disease could arise from hypertension.[38] The direction of the relationship between blood pressure and kidney function, has been the subject of debate.[39] However, evidence from genetic studies suggests that the association between renal function and blood pressure is likely to likely to be explained by decreased renal function giving rise to high blood pressure. In a large (n>200,000) genome wide association study (GWAS), loci that were associated with blood pressure elevation and cardiovascular disease showed no association with kidney disease or kidney function.[29] If SCT compromises renal function and this in turn leads to elevated blood pressure, this would result in a bias toward a null result when using SCT as a proxy for testing the malaria-high blood pressure hypothesis. As can be seen in Figure S3, impaired kidney function (as measured by eGFR) is associated with both the exposure and the outcome, but is not on the causal pathway from malaria to the outcome. Kidney function is therefore considered a confounder and we adjusted for eGFR in all regression analyses. We also examined the effect of using urine albumin to creatinine ratio in place of eGFR in the regression models.

If, however, renal function lies on the causal pathway between malaria and high blood pressure it would be inappropriate to include it within the regression models. Severe malaria does occasionally present with acute renal failure and repeated episodes of malaria could result in chronic pyelonephritis and elevated BP. However, acute renal failure is a very rare complication of malaria in Kilifi, for example, it occurred in 2 out of 1844 children admitted with malaria.[40] This suggests that renal failure is an unlikely mediator of the potential association between malaria and elevated BP.

We confirmed that each of the *a priori* specified covariates (age, sex and eGFR) significantly improved the regression models using the likelihood ratio test.

Confounding due to pleiotropy

A special type of confounding can also occur if the genetic trait influences the outcome through a pathway that is independent of the exposure (pleiotropy)[41] as illustrated in Figure S4.

In contrast with renal function, which is a known confounder and can be measured and adjusted for in regression analyses, confounding due to other (often unknown) causes can only be detected by examining the relationship between sickle cell trait and blood pressure in individuals who have not been exposed to malaria. The existence of pleiotropy can invalidate the use of the genetic trait as a marker for the infectious disease exposure. In order to exclude pleiotropy as a potential explanation for the association between SCT and BP, we studied lifelong residents of Nairobi, Kenya and Jackson, Mississippi, two sites where there is no malaria transmission. In addition, we conducted a pooled analysis incorporating data from the three study sites and conducted a linear regression with the previously specified covariates plus SCT and study site and their interaction. This increased the power to detect any

independent effect of SCT on BP while simultaneously checking for differential effect of SCT according to study site.

ii) Effect modifier: α ⁺thalassemia

α^{+}thalassemia, in which there is reduction in the amount of alpha hemoglobin, is common in regions where malaria transmission occurs. Williams et al[42] have demonstrated a negative epistatic effect when α+thalassemia is coinherited with SCT. The effect of coinheritance of the mutations is to reduce the malaria protective effect of SCT to about 27% (from 50%) for uncomplicated malaria and to 44% (from 80%) for severe malaria.[42] Put simply, the presence of α^{+}thalassemia reduces the protective effect of SCT against both uncomplicated and severe malaria by about half. We therefore included α^{+}thalassemia as an interaction term (interacting with SCT) in the main regression model and examined whether its inclusion changed the effect estimate for SCT in predicting blood pressure.

In a related analysis, we ran a linear regression model examining the effect of α^{+}thalassemia on blood pressure with the same covariates used in the main analysis for SCT. Because α^{+}thalassemia confers less protection against malaria than SCT, we expected that the effect estimate in this model would be lower than that of SCT.

f) Testing for cohort effects

Malaria incidence in Kilifi has been changing over time and we considered that this could influence results obtained. Data on the changing levels of transmission go back to 1990 and they show that a significant drop in transmission in Kilifi began around 1999-2000.[43] In addition, because blood pressure rises with age, it is possible that the effects of malaria on outcome measures may be more apparent later in life. While it is not possible to determine the individual contributions of

changing malaria exposure and aging to any differences observed in outcome measures, we attempted to display these differences by performing comparisons of the outcomes by sickle trait in 3 age strata.

Table S1. Characteristics of those who consented to undergo ABPM versus those who declined.

	Kilifi N=2537					Nairobi N=1119					Jackson N=5306				
	Consented n=2371		Did not consent n=166			Consented n=1026		Did not consent n=93			Consented n=1148		Did not consent n=4158		
Characteristic	n	*(%)*	n	*(%)*	p-value	n	*(%)*	n	*(%)*	p-value	n	*(%)*	n	*(%)*	p-value
Female	1361	*(54)*	84	*(51)*	0.408	480	*(47)*	45	*(48)*	0.443	780	*(68)*	2591	*(62)*	<0.001
	mean	*(SD)*	mean	*(SD)*	p-value	mean	*(SD)*	mean	*(SD)*	p-value	mean	*(SD)*	mean	*(SD)*	p-value
Mean Age, years	39	*(22)*	48	*(22)*	<0.001	22	*(16)*	25	*(17)*	0.073	59	*(11)*	54	*(13)*	<0.001

Table S2. Characteristics of participants with and without good quality ABPM data.

	Kilifi N=2371					Nairobi N=1026					Jackson N=1148				
	Met ESH criteria n=1140		Did not meet ESH criteria n=1231		p-value	Met ESH criteria n=542		Did not meet ESH criteria n=484		p-value	Met ESH criteria n=934		Did not meet ESH criteria n=214		p-value
Characteristic	n	*(%)*	n	*(%)*		n	*(%)*	n	*(%)*		n	*(%)*	n	*(%)*	
Female	660	*(58)*	520	*(48)*	<0.001	290	*(54)*	237	*(49)*	0.300	630	*(67)*	150	*(73)*	0.134
Have sickle cell trait	240	*(21)*	205	*(19)*	0.240	83	*(15)*	50	*(14)*	0.637	58	*(8.9)*	10	*(7.5)*	0.588
Smoker	96	*(8)*	87	*(8)*	0.761	11	*(2)*	12	*(3)*	0.198	103	*(11)*	40	*(19)*	0.001
Previously diagnosed hypertensive[§]	165	*(14)*	133	*(12)*	0.140	62	*(11)*	23	*(7)*	0.017	560	*(60)*	122	*(59)*	0.779
On anti-hypertensive medication	36	*(3)*	16	*(1.5)*	0.009	9	*(2)*	3	*(1)*	0.315	519	*(59)*	116	*(59)*	0.930
	mean	*(SD)*	mean	*(SD)*	p-value	mean	*(SD)*	mean	*(SD)*	p-value	mean	*(SD)*	mean	*(SD)*	p-value
Age, years	40	*(22)*	36	*(21)*	<0.001	23	*(17)*	19	*(15)*		59	*(11)*	58	*(12)*	0.360
BMI kg/m^2	20.6	*(3.8)*	20.6	*(4.0)*	0.689	20.4	*(4.1)*	19.7	*(4.0)*	0.010	30.9	*(6.5)*	33.1	*(5.8)*	<0.001
HbA1c, %	5.1	*(0.7)*	5.1	*(0.6)*	0.134	5.3	*(0.98)*	5.4	*(1.2)*	0.043	6.0	*(1.3)*	6.1	*(1.4)*	0.776
Hemoglobin, g/dl	12.6	*(1.7)*	12.9	*(1.6)*	<0.001	13.3	*(1.7)*	13.4	*(1.7)*	0.421	13.0	*(1.4)*	13.0	*(1.5)*	0.661
WBC count X10^9/L	5.7	*(1.4)*	5.7	*(1.6)*	0.718	5.4	*(1.6)*	5.5	*(1.4)*	0.187	5.3	*(1.6)*	5.8	*(1.9)*	0.002
Platelet count X10^9/L	264	*(86)*	257	*(81)*	0.064	289	*(100)*	302	*(88)*	0.046	243	*(62)*	250	*(69)*	0.078
eGFR, ml/min/1.73m^2	113	*(41)*	119	*(40)*	<0.001	116	*(25)*	119	*(24)*	0.090	86.6	*(25)*	87.8	*(25)*	0.540
Log UACR, mg/g	1.2	*(0.62)*	1.2	*(0.61)*	0.674	1.3	*(0.71)*	1.40	*(0.60)*	0.345	0.90	*(0.50)*	0.90	*(0.56)*	0.860

ABPM: Ambulatory blood pressure monitoring; eGFR: Estimated glomerular filtration rate; ESH: European society of hypertension. UACR: urine albumin to creatinine ratio
ESH criteria require a minimum of 20 valid readings taken between 9 a.m. and 9 p.m. and a minimum of 7 valid readings taken between 1a.m. and 6 a.m. in order for ABPM readings to be considered as complete.
[§]Answered "yes" to the question: Has a doctor or healthcare worker previously told you that you have high blood pressure?

Table S3. Effect of sickle cell trait on blood pressure in Nairobi and Jackson.

	Nairobi (N=516)			Jackson (N=651)			
ABPM measure	β (mm Hg)	*(95% CI)*	p value	β (mm Hg)	*(95% CI)*	p value	p-value for interaction
24 hour SBP	0.6	*(–2.5, 3.7)*	0.722	0.6	*(–3.0, 4.2)*	0.732	0.944
24 hour DBP	0.5	*(–1.6, 2.6)*	0.652	0.8	*(–1.5, 3.1)*	0.489	0.695
Nighttime SBP	0.7	*(–2.5, 4.0)*	0.669	–0.2	*(–4.3, 4.0)*	0.938	0.766
Nighttime DBP	0.6	*(–1.7, 2.9)*	0.610	0.8	*(–1.8, 3.3)*	0.558	0.779
Daytime SBP	–0.1	*(–3.4, 3.2)*	0.963	1.3	*(–2.3, 4.9)*	0.486	0.567
Daytime DBP	0.1	*(–2.3, 2.4)*	0.951	1.2	*(–1.2, 3.6)*	0.310	0.409

Linear regression models adjusted for age, sex and estimated glomerular filtration rate
SBP: Systolic blood pressure
DBP: Diastolic blood pressure
p-value for interaction represents result of regression models testing for difference in effect of sickle cell trait on BP in Nairobi versus Jackson

Table S4. Effects of sickle cell trait on blood pressure: effect of excluding participants taking anti-hypertensive medication.

All participants

	Kilifi (N=1127)			Nairobi and Jackson (N=1166)		
ABPM measure	β (mm Hg)	*(95% CI)*	p value	β (mm Hg)	*(95% CI)*	p value
24 hour SBP	–2.4	*(–4.7, –0.2)*	0.037	0.7	*(–1.6, 3.1)*	0.542
24 hour DBP	–1.4	*(–2.8, 0.1)*	0.068	0.1	*(–1.5, 1.8)*	0.860
Nighttime SBP	–3.2	*(–5.7, –0.6)*	0.015	0.5	*(–2.2, 3.1)*	0.727
Nighttime DBP	–1.8	*(–3.3, –0.2)*	0.026	0.3	*(–1.5, 2.0)*	0.773
Daytime SBP	–1.9	*(–4.2, 0.4)*	0.113	0.7	*(–1.7, 3.1)*	0.566
Daytime DBP	–1.0	*(–2.6, 0.6)*	0.223	0.1	*(–1.6, 1.8)*	0.889

Excluding participants on medication

	Kilifi (N=1092)			Nairobi and Jackson (N=755)		
ABPM measure	β (mm Hg)	*(95% CI)*	p value	β (mm Hg)	*(95% CI)*	p value
24 hour SBP	–2.5	*(–4.8, –0.2)*	0.034	0.2	*(–2.5, 2.8)*	0.896
24 hour DBP	–1.4	*(–2.8, 0.1)*	0.067	–0.2	*(–2.0, 1.5)*	0.787
Nighttime SBP	–3.1	*(–5.7, –0.6)*	0.017	0.8	*(–2.1, 3.6)*	0.597
Nighttime DBP	–1.8	*(–3.3, –0.2)*	0.028	0.1	*(–1.8, 2.1)*	0.902
Daytime SBP	–2.0	*(–4.3, 0.4)*	0.096	–0.4	*(–3.1, 2.4)*	0.802
Daytime DBP	–1.0	*(–2.6, 0.6)*	0.205	–0.5	*(–2.4, 1.5)*	0.630

Linear regression models adjusted for age, sex and estimated glomerular filtration rate
SBP: Systolic blood pressure
DBP: Diastolic blood pressure

Table S5. Effect of Sickle cell trait on blood pressure by sex and study site.

Kilifi

	Women (N=659)			Men (N=473)			
ABPM measure	β	*(95% CI)*	p value	β	*(95% CI)*	p value	Interaction p-value
24 hour SBP	–3.7	*(–7.1, –0.4)*	0.028	–0.7	*(–3.7, 2.3)*	0.645	0.218
24 hour DBP	–1.9	*(–2.8, 0.1)*	0.079	–0.7	*(–2.7, 1.3)*	0.493	0.398
Nighttime SBP	–4.3	*(–8.0, –0.6)*	0.022	–1.6	*(–5.1, 1.8)*	0.356	0.335
Nighttime DBP	–2.0	*(–4.2, 0.1)*	0.067	–1.4	*(–3.6, 0.8)*	0.217	0.661
Daytime SBP	–3.1	*(–6.4, 0.2)*	0.068	–0.3	*(–3.4, 2.9)*	0.875	0.284
Daytime DBP	–1.6	*(–3.9, 0.6)*	0.154	–0.2	*(–2.4, 2)*	0.867	0.131

Nairobi and Jackson

	Women (N=705)			Men (N=461)			
ABPM measure	β	*(95% CI)*	p value	β	*(95% CI)*	p value	Interaction p-value
24 hour SBP	0.9	*(–2.2, 4)*	0.574	0.7	*(–2.9, 4.4)*	0.700	0.800
24 hour DBP	1.2	*(–0.9, 3.3)*	0.272	–0.4	*(–2.9, 2.1)*	0.737	0.106
Nighttime SBP	1.2	*(–2.3, 4.7)*	0.511	–0.2	*(–4.2, 3.8)*	0.915	0.512
Nighttime DBP	1.7	*(–0.6, 3.9)*	0.139	–0.8	*(–3.5, 2.0)*	0.586	0.046
Daytime SBP	0.7	*(–2.5, 3.9)*	0.679	1.0	*(–2.8, 4.8)*	0.617	0.998
Daytime DBP	0.8	*(–1.4, 3.1)*	0.464	–0.1	*(–2.7, 2.5)*	0.936	0.319

Linear regression models adjusted for age, sex and estimated glomerular filtration rate
SBP: Systolic blood pressure
DBP: Diastolic blood pressure
p-value for interaction represents result of regression models testing for difference in effect of sickle cell trait on BP in men versus women

Table S6. Results of interaction analysis comparing effect of sickle cell trait in Kilifi versus Nairobi/Jackson using log urine albumin to creatinine ratio as covariate instead of estimated Glomerular Filtration Rate.

	All (N=1583)			Women N=958			Men N=625		
	β	*(95% CI)*	p value	β	*(95% CI)*	p value	β	*(95% CI)*	p value
24 hour SBP	–5.2	*(–9.5, –0.9)*	0.019	–6.7	*(–13, –0.6)*	0.030	–3.5	*(–9.5, 2.5)*	0.249
24 hour DBP	–2.9	*(–5.7, –0.1)*	0.040	–4.6	*(–8.5, –0.8)*	0.019	–1.0	*(–5.0, 3.1)*	0.635
Nighttime SBP	–5.5	*(–10, –0.7)*	0.026	–6.3	*(–13, 0.5)*	0.067	–4.7	*(–12, 2.1)*	0.176
Nighttime DBP	–3.6	*(–6.6, –0.6)*	0.018	–5.1	*(–9.1, –1.1)*	0.013	–1.7	*(–6.2, 2.8)*	0.456
Daytime SBP	–4.8	*(–9.2, –0.4)*	0.032	–6.5	*(–13, –0.4)*	0.037	–3.2	*(–9.5, 3.0)*	0.308
Daytime DBP	–2.5	*(–5.5, 0.5)*	0.106	–4.1	*(–8.3, 0.03)*	0.052	–0.9	*(–5.3,3.4)*	0.673

SCT: sickle cell trait
SBP: Systolic blood pressure
DBP: Diastolic blood pressure
Linear regression models tested for interaction in the effect of SCT by site. Other covariates were age, sex and log urine albumin to creatinine ratio

Table S7. Results of pooled analysis comparing effect of sickle cell trait in Kilifi versus Nairobi/Jackson stratified by sex.

	Women (N=1359)			Men (N=934)			
ABPM measure	β	*(95% CI)*	p value	β	*(95% CI)*	p value	Interaction p-value
24 hour SBP	–5.0	*(–9.7, –0.2)*	0.039	–1.9	*(–6.6, 2.8)*	0.431	0.419
24 hour DBP	–3.2	*(–6.2, –0.1)*	0.041	–0.3	*(–3.5, 2.9)*	0.871	0.110
Nighttime SBP	–5.8	*(–11, –0.6)*	0.030	–1.8	*(–7.1, 3.5)*	0.506	0.323
Nighttime DBP	–3.9	*(–7.1, –0.7)*	0.017	–0.5	*(–4.0, 3.0)*	0.782	0.086
Daytime SBP	–4.1	*(–8.9, 0.7)*	0.091	–1.8	*(–6.7, 3.2)*	0.489	0.549
Daytime DBP	–2.5	*(–5.8, 0.7)*	0.126	–0.2	*(–3.6, 3.3)*	0.925	0.207

Estimates were derived separately for each sex as the interaction term (malaria vs non-malaria sites) in a linear regression of blood pressure by sickle cell trait status after adjusting for age and estimated glomerular filtration rate. Interaction p-value is the result of 3-way interaction in regression models testing for difference in effect of sickle cell trait on BP in men versus women in Kilifi versus Nairobi and Jackson pooled together. DBP: diastolic blood pressure; SBP: systolic blood pressure.

Table S8. Effect of α^+thalassemia on ambulatory blood pressure by study site

Kilifi (N=1125).

	No. of –3.7kb α^+ thalassemia deletions					
	One deletion (Heterozygous)			Two deletions (Homozygous)		
ABPM measure	β mm Hg	*(95% CI)*	p value	β mm Hg	*(95% CI)*	p value
24 hour SBP	–0.1	*(–2.2, 2.0)*	0.921	–1.1	*(–3.8, 1.6)*	0.434
24 hour DBP	–0.3	*(–1.7, 1.0)*	0.651	–0.4	*(–2.2, 1.3)*	0.646
Nighttime SBP	0.2	*(–2.2, 2.6)*	0.879	–1.3	*(–4.3, 1.8)*	0.416
Nighttime DBP	–0.1	*(–2.3, 1.4)*	0.919	–0.5	*(–2.4, 1.4)*	0.597
Daytime SBP	–0.2	*(–2.3, 2.0)*	0.884	–1.0	*(–3.7, 1.8)*	0.500
Daytime DBP	–0.1	*(–1.5, 1.4)*	0.930	–0.3	*(–2.2, 1.6)*	0.765

Nairobi (N=514)

	No. of –3.7kb α^+ thalassemia deletions					
	One deletion (Heterozygous)			Two deletions (Homozygous)		
ABPM measure	β mm Hg	*(95% CI)*	p value	β mm Hg	*(95% CI)*	p value
24 hour SBP	1.1	*(–1.3, 3.5)*	0.373	3.9	*(–0.5, 8.2)*	0.083
24 hour DBP	0.6	*(–1.0, 2.3)*	0.440	2.6	*(–0.4, 5.6)*	0.086
Nighttime SBP	1.9	*(–0.6,4.4)*	0.144	3.3	*(–1.3, 7.9)*	0.157
Nighttime DBP	1.8	*(0.05, 3.6)*	0.044	2.4	*(–0.8, 5.6)*	0.138
Daytime SBP	0.04	*(–1.8, 2.6)*	0.974	2.9	*(–1.8, 7.5)*	0.223
Daytime DBP	–0.3	*(–2.1, 1.5)*	0.748	2.2	*(–1.0, 5.5)*	0.181

SBP: Systolic blood pressure
DBP: Diastolic blood pressure
Linear regression models adjusted for age, sex and estimated glomerular filtration rate.
*No alpha thalassemia data were available for participants from Jackson

Results of analyses based on data meeting IDACO criteria for completeness

Table S9. Characteristics of study participants with and without sickle cell trait by study site (IDACO Criteria).

	Kilifi N=2048				Nairobi N=835				Jackson N=724			
	SCT n=408		Non-SCT n=1640		SCT n=121		Non-SCT n=714		SCT n=63		Non-SCT n=661	
Characteristic	n	*(%)*	n	*(%)*	n	*(%)*	n	*(%)*	n	*(%)*	n	*(%)*
Female	203	*(50)*	884	*(54)*	59	*(49)*	369	*(52)*	41	*(65)*	439	*(66)*
Smoker	34	*(8)*	134	*(8)*	5	*(4)*	17	*(2)*	9	*(14)*	83	*(13)*
Previously diagnosed hypertensive[§]	59	*(15)*	221	*(14)*	9	*(7)*	70	*(10)*	33	*(52)*	408	*(62)*
Taking antihypertensive medication	11	*(3)*	38	*(2)*	2	*(2)*	10	*(1)*	27	*(44)*	377	*(61)*
	mean	*(SD)*	mean	*(SD)*	mean	*(SD)*	mean	*(SD)*	mean	*(SD)*	mean	*(SD)*
Age, years	40	*(22)*	38	*(22)*	20	*(14)*	22	*(16)*	60	*(12)*	60	*(11)*
BMI kg/m^2	20.7	*(3.8)*	20.7	*(3.9)*	20.2	*(4.0)*	20.2	*(4.1)*	31.0	*(6.9)*	31.1	*(6.4)*
HbA1c, %	5.2	*(0.6)*	5.1	*(0.7)*	5.4	*(1.3)*	5.3	*(1.0)*	6.0	*(1.2)*	6.1	*(1.3)*
Hemoglobin, g/dl	12.7	*(1.9)*	12.7	*(1.6)*	13.4	*(1.8)*	13.3	*(1.7)*	12.8	*(1.4)*	13.0	*(1.4)*
WBC count X10^9/L	5.8	*(1.5)*	6.5	*(22.5)*	5.4	*(1.3)*	5.4	*(1.5)*	4.9	*(1.2)*	5.3	*(1.5)*
Platelet count X10^9/L	261	*(89)*	259	*(81)*	289	*(96)*	294	*(97)*	229	*(57)*	243	*(62)*
Plasma osmolality, mosm/Kg	290	*(5.8)*	290	*(6.3)*	291	*(12)*	291	*(12)*	-	-	-	-
eGFR, ml/min/1.73m^2	111	*(39)*	116	*(40)*	117	*(27)*	116	*(24)*	84	*(27)*	87	*(26)*
Log UACR, mg/g	1.3	*(0.6)*	1.2	*(0.6)*	1.5	*(0.7)*	1.3	*(0.7)*	1.1	*(0.6)*	0.9	*(0.5)*

BMI: Body mass index; eGFR: estimated glomerular filtration rate; HbA1c: glycosylated hemoglobin; SCT: Sickle cell trait; SD: standard deviation; UACR: urine albumin to creatinine ratio; WBC: white blood cell. Plasma osmolality measurements were not available for Jackson participants.

[§]Answered “yes” to the question: Has a doctor or healthcare worker previously told you that you have high blood pressure?

Table S10. Effect of sickle cell trait on blood pressure by malaria site (IDACO Criteria).

	Kilifi (N=1965)			Nairobi and Jackson (N=1500)			Nairobi/Jackson Interaction
ABPM measure	β (mmHg)	*(95% CI)*	p value	β (mm Hg)	*(95% CI)*	p value	p-value for interaction
24 hour SBP	–1.4	*(–3.1, 0.4)*	0.128	0.4	*(–1.6, 2.5)*	0.697	0.557
24 hour DBP	–0.7	*(–1.8, 0.4)*	0.226	0.1	*(–1.3, 1.5)*	0.910	0.394
Nighttime SBP	–1.6	*(–3.5, 0.4)*	0.109	–0.4	*(–2.6, 1.9)*	0.744	0.622
Nighttime DBP	–0.9	*(–2.1, 0.3)*	0.146	–0.3	*(–1.8, 1.2)*	0.685	0.273
Daytime SBP	–0.9	*(–2.7, 0.9)*	0.327	0.6	*(–1.6, 2.7)*	0.602	0.264
Daytime DBP	–0.6	*(–1.8, 0.7)*	0.367	0.1	*(–1.4, 1.6)*	0.858	0.237

Linear regression models adjusted for age, sex and estimated glomerular filtration rate
SBP: Systolic blood pressure
DBP: Diastolic blood pressure
p-value for interaction represents result of regression models testing for difference in effect of sickle cell trait on BP in Nairobi versus Jackson

Table S11. Age specific effects of sickle cell trait on blood pressure by study site (IDACO Criteria).

Kilifi

		24 hour blood pressure				Night time blood pressure				Daytime blood pressure			
Age, years	N	SBP	*(95% CI)*	DBP	*(95% CI)*	SBP	*(95% CI)*	DBP	*(95% CI)*	SBP	*(95% CI)*	DBP	*(95% CI)*
10-29	917	–0.7	*(–2.5,1.1)*	0.2	*(–1.0, 1.4)*	–0.2	*(–2.2,1.7)*	0.4	*(–0.9, 1.7)*	–1.5	*(–3.5, 0.6)*	0	*(–1.4, 1.4)*
30-59	655	–3.0	*(–6.3, 0.3)*	–2.3	*(–4.5, –0.1)*	–3.8	*(–7.5, –0.2)*	–2.8	*(–5.1, 0.5)*	–1.5	*(–4.8, 1.9)*	–1.8	*(–4.2, 0.5)*
≥60	393	–0.2	*(–5.3, 4.9)*	–0.4	*(–3.1, 3.0)*	–0.8	*(–6.6, 4.9)*	–0.5	*(–3.7, 2.7)*	1.0	*(–4.0, 6.1)*	0.3	*(–2.8, 3.6)*

Nairobi and Jackson pooled together±

Age, years	N	SBP	*(95% CI)*	DBP	*(95% CI)*	SBP	*(95% CI)*	DBP	*(95% CI)*	SBP	*(95% CI)*	DBP	*(95% CI)*
10-29	620	–0.5	*(–3.0, 1.9)*	0	*(–1.5. 1.5)*	–1.1	*(–3.6, 1.4)*	–0.6	*(–2.2, 1.1)*	–0.9	*(–3.6, 1.8)*	–0.4	*(–2.2, 1.5)*
30-59	451	–0.4	*(–4.6, 3.9)*	–1.2	*(–4.0, 1.6)*	–0.7	*(–5.5, 4.0)*	–0.6	*(–3.7, 2.5)*	–0.2	*(–4.5, 4.1)*	–0.9	*(–3.9, 2.1)*
≥60	429	3.7	*(–1.2, 8.6)*	4.0	*(0.9, 7.0)*	2.0	*(–3.5, 7.6)*	3.1	*(–0.2, 6.4)*	5.1	*(0.1, 10.0)*	4.8	*(1.5, 8.0)*

SBP: Systolic blood pressure
DBP: Diastolic blood pressure
Results of linear regression models adjusted for age, sex and estimated glomerular filtration rate
±Participants in Jackson were aged 21 years and older

Table S12. Effect of Sickle cell trait on blood pressure by sex and study site (IDACO Criteria).

Kilifi

	Women (N=1046)			Men (N=919)			
ABPM measure	β	*(95% CI)*	p value	β	*(95% CI)*	p value	Interaction p-value
24 hour SBP	–1.9	*(–4.5, 0.6)*	0.141	–0.8	*(–3.2, 1.7)*	0.537	0.533
24 hour DBP	–0.7	*(–2.3, 0.9)*	0.396	–0.7	*(–2.3, 0.8)*	0.362	0.910
Nighttime SBP	–2.0	*(–4.9, 0.8)*	0.160	–1.1	*(–3.7, 1.6)*	0.423	0.653
Nighttime DBP	–0.7	*(–2.4, 1.0)*	0.417	–1.1	*(–2.8, 0.6)*	0.200	0.825
Daytime SBP	–1.4	*(–3.9, 1.2)*	0.292	–0.4	*(–3.0, 2.2)*	0.759	0.612
Daytime DBP	–0.8	*(–2.5, 0.9)*	0.353	–0.4	*(–2.1, 1.4)*	0.679	0.620

Nairobi and Jackson

	Women (N=879)			Men (N=621)			
ABPM measure	β	*(95% CI)*	p value	β	*(95% CI)*	p value	Interaction p-value
24 hour SBP	0.1	*(–2.7, 2.8)*	0.962	1.0	*(–2.1, 4.0)*	0.525	0.272
24 hour DBP	0.7	*(–1.1, 2.6)*	0.429	–0.3	*(–2.3, 1.8)*	0.807	0.371
Nighttime SBP	–0.7	*(–3.7, 2.4)*	0.674	0.1	*(–3.1, 3.4)*	0.930	0.294
Nighttime DBP	0.5	*(–1.4, 2.5)*	0.604	–0.8	*(–3.1, 1.4)*	0.476	0.393
Daytime SBP	–0.3	*(–3.1, 2.6)*	0.856	1.8	*(–1.4, 5.0)*	0.275	0.096
Daytime DBP	0.2	*(–1.8, 2.1)*	0.881	0.6	*(–1.7, 2.8)*	0.612	0.110

Linear regression models adjusted for age, sex and estimated glomerular filtration rate
SBP: Systolic blood pressure
DBP: Diastolic blood pressure
p-value for interaction represents result of regression models testing for difference in effect of sickle cell trait on BP in men versus women

Table S13. Effects of sickle cell trait on blood pressure: effect of excluding participants taking anti-hypertensive medication (IDACO Criteria).

	Kilifi (N=1918)			Nairobi and Jackson (N=1041)		
ABPM measure	β (mm Hg)	*(95% CI)*	p value	β (mm Hg)	*(95% CI)*	p value
24 hour SBP	–1.3	*(–3.1, 0.5)*	0.157	–0.3	*(–2.5, 1.9)*	0.790
24 hour DBP	–0.7	*(–1.8, 0.5)*	0.254	–0.4	*(–1.9, 1.1)*	0.613
Nighttime SBP	–1.5	*(–3.4, 0.5)*	0.136	–0.6	*(–2.9, 1.8)*	0.639
Nighttime DBP	–0.9	*(–2.0, 0.3)*	0.162	–0.7	*(–2.3, 0.8)*	0.356
Daytime SBP	–0.9	*(–2.7, 1.0)*	0.348	–0.4	*(–2.8, 2.0)*	0.729
Daytime DBP	–0.5	*(–1.8, 0.7)*	0.382	–0.4	*(–2.0, 1.3)*	0.678

Table S14. Results of interaction analysis comparing effect of sickle cell trait in Kilifi versus Nairobi/Jackson (IDACO Criteria).

	Model 1 (N=3465)			Model 2 N=2379		
	β	*(95% CI)*	p value	β	*(95% CI)*	p value
24 hour SBP	–2.1	*(–5.0, 0.7)*	0.146	–3.3	*(–7.0, 0.4)*	0.078
24 hour DBP	–1.0	*(–2.9, 0.8)*	0.275	–2.3	*(–4.7, 0.1)*	0.057
Nighttime SBP	–1.5	*(–4.6, 1.6)*	0.339	–3.1	*(–7.2, 0.9)*	0.133
Nighttime DBP	–0.8	*(–2.8, 1.1)*	0.337	–2.7	*(–5.3, –0.2)*	0.032
Daytime SBP	–1.8	*(–4.8, 1.1)*	0.225	–3.1	*(–6.8, 0.7)*	0.112
Daytime DBP	–0.9	*(–2.9, 1.1)*	0.394	–2.0	*(–4.6, 0.6)*	0.126

Estimates were derived as the interaction term (malaria vs non-malaria sites) in a linear regression of blood pressure by sickle cell trait status after adjusting for age, sex and renal function. Renal function was represented by estimated glomerular filtration rate in model 1 and by log urine albumin to creatinine ratio in model 2. SBP: Systolic blood pressure. DBP: Diastolic blood pressure

Table S15. Effect of α^{+}thalassemia on ambulatory blood pressure by study site (IDACO Criteria).

Kilifi (N=1961)

	No. of –3.7kb α^{+} thalassemia deletions					
	One deletion (Heterozygous)			Two deletions (Homozygous)		
ABPM measure	β mm Hg	*(95% CI)*	p value	β mm Hg	*(95% CI)*	p value
24 hour SBP	–0.4	*(–1.9, 1.2)*	0.637	–1.9	*(–3.9, 0.2)*	0.073
24 hour DBP	–0.4	*(–1.4, 0.7)*	0.480	–0.7	*(–2.0, 0.6)*	0.286
Nighttime SBP	–0.1	*(–1.9, 1.6)*	0.879	–1.5	*(–3.7, 0.8)*	0.203
Nighttime DBP	–0.3	*(–1.4, 0.8)*	0.605	–0.6	*(–2.0, 0.8)*	0.397
Daytime SBP	–0.5	*(–2.1, 1.2)*	0.581	–2.1	*(–4.1, 0.1)*	0.059
Daytime DBP	–0.1	*(–1.2, 1.0)*	0.808	–0.7	*(–2.1, 0.8)*	0.361

Nairobi (N=771)

	No. of –3.7kb α^{+} thalassemia deletions					
	One deletion (Heterozygous)			Two deletions (Homozygous)		
ABPM measure	β mm Hg	*(95% CI)*	p value	β mm Hg	*(95% CI)*	p value
24 hour SBP	0.7	*(–1.3, 2.6)*	0.494	1.6	*(–2.0, 5.2)*	0.390
24 hour DBP	0.4	*(–0.6, 1.6)*	0.572	1.4	*(—0.9, 3.7)*	0.238
Nighttime SBP	0.9	*(–1.1, 3.0)*	0.376	1.2	*(–2.6, 4.9)*	0.376
Nighttime DBP	1.0	*(–0.4, 2.3)*	0.164	1.0	*(–1.4, 3.5)*	0.420
Daytime SBP	0.1	*(–3.7, 4.1)*	0.941	0.2	*(–3.7, 4.1)*	0.923
Daytime DBP	–0.2	*(–1.6, 1.3)*	0.837	0.5	*(–2.2, 3.1)*	0.717

SBP: Systolic blood pressure
DBP: Diastolic blood pressure
Linear regression models adjusted for age, sex and estimated glomerular filtration rate.
*No alpha thalassemia data were available for participants from Jackson

Figure S1. Study locations.

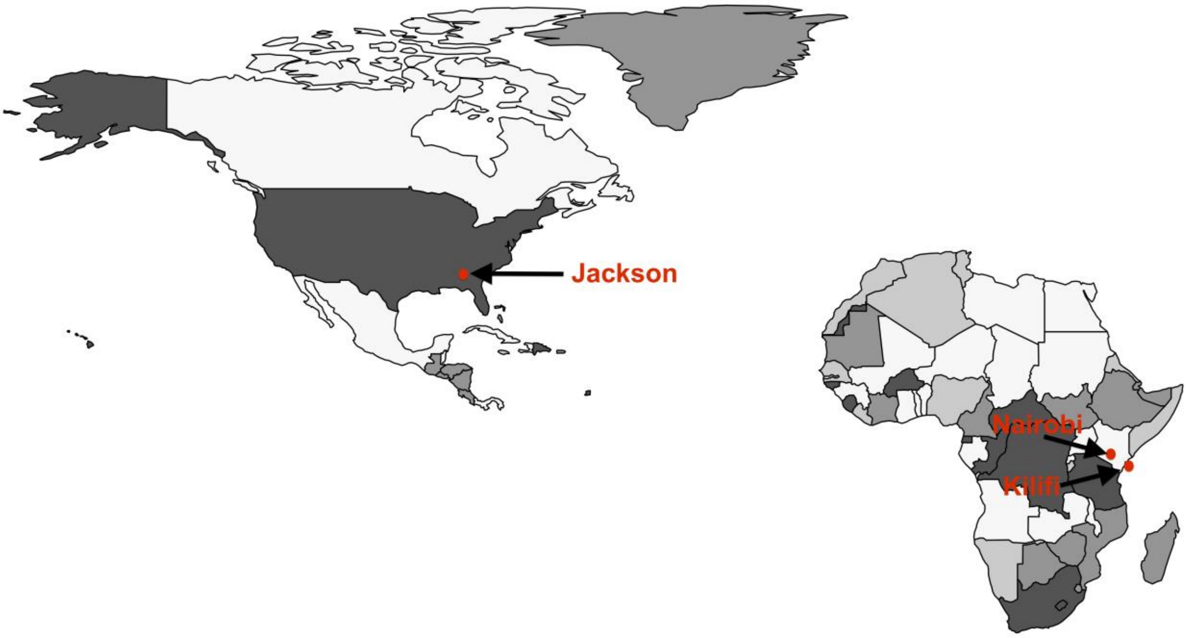

Figure S2. Causal diagram for the malaria high blood pressure hypothesis.

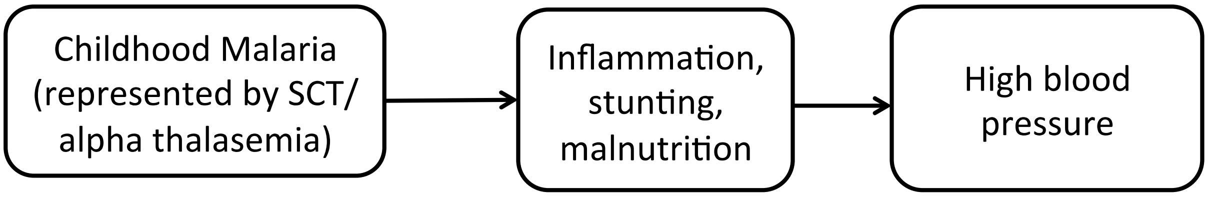

Figure S3. Illustrating confounding effect of kidney function (eGFR) in individuals with SCT.

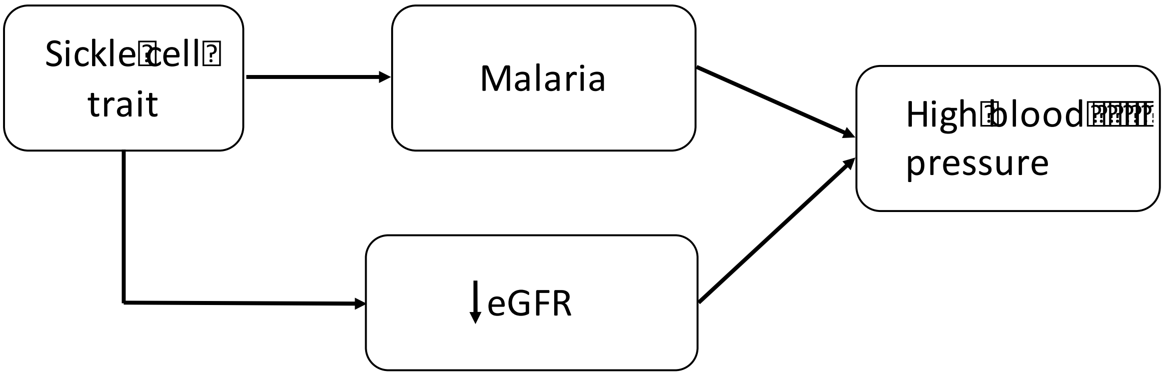

Figure S4. Illustrating confounding due to pleiotropy.

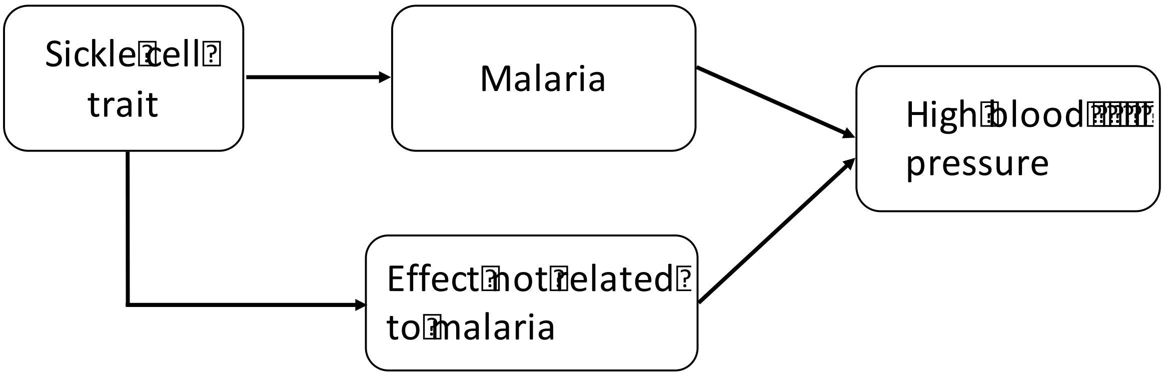

Supplemental References:

1. Beguy D, Elung'ata P, Mberu B, Oduor C, Wamukoya M, Nganyi B and Ezeh A. Health & Demographic Surveillance System Profile: The Nairobi Urban Health and Demographic Surveillance System (NUHDSS). *Int J Epidemiol*. 2015;44:462-71.

2. Scott JA, Bauni E, Moisi JC, Ojal J, Gatakaa H, Nyundo C, Molyneux CS, Kombe F, Tsofa B, Marsh K, Peshu N and Williams TN. Profile: The Kilifi Health and Demographic Surveillance System (KHDSS). *Int J Epidemiol*. 2012;41:650-7.

3. Flint J, Hill AV, Bowden DK, Oppenheimer SJ, Sill PR, Serjeantson SW, Bana-Koiri J, Bhatia K, Alpers MP, Boyce AJ and et al. High frequencies of alpha-thalassaemia are the result of natural selection by malaria. *Nature*. 1986;321:744-50.

4. Foy H, Ph D, Sc D, Kondi A, Bushra F, Hall F and Bari B-s. The variability of sickle cell rates in the tribes of Kenya and the Southern Sudan. *BMJ*. 1954;1:294-297.

5. Etyang AO, Warne B, Kapesa S, Munge K, Bauni E, Cruickshank JK, Smeeth L and Scott JA. Clinical and Epidemiological Implications of 24-Hour Ambulatory Blood Pressure Monitoring for the Diagnosis of Hypertension in Kenyan Adults: A Population-Based Study. *J Am Heart Assoc*. 2016;5:e004797-e004797.

6. Alkire S and Foster J. Understandings and misunderstandings of multidimensional poverty measurement. *The Journal of Economic Inequality*. 2011;9:289-314.

7. Nemeth Z, Moczar K and Deak G. Evaluation of the Tensioday ambulatory blood pressure monitor according to the protocols of the British Hypertension Society and the Association for the Advancement of Medical Instrumentation. *Blood Press Monit*. 2002;7:191-7.

8. Narayanan S and Appleton HD. Creatinine: a review. *Clin Chem*. 1980;26:1119-26.

9. Levey AS, Stevens LA, Schmid CH, Zhang YL, Castro AF, 3rd, Feldman HI, Kusek JW, Eggers P, Van Lente F, Greene T, Coresh J and Ckd EPI. A new equation to estimate glomerular filtration rate. *Ann Intern Med*. 2009;150:604-12.

10. Schwartz GJ, Munoz A, Schneider MF, Mak RH, Kaskel F, Warady BA and Furth SL. New equations to estimate GFR in children with CKD. *J Am Soc Nephrol*. 2009;20:629-37.

11. Taylor HA, Jr. The Jackson Heart Study: an overview. *Ethn Dis*. 2005;15:S6-1-3.

12. Wilson JG, Rotimi CN, Ekunwe L, Royal CDM, Crump ME, Wyatt SB, Steffes MW, Adeyemo A, Zhou J, Pharm D and Jr HAT. Study Design For Genetics In The Jackson Heart Study. *Ethn Dis*. 2005;15:S6-30 - S6-37.

13. Carpenter MA, Crow R, Steffes M, Rock W, Heilbraun J, Evans G, Skelton T, Jensen R and Sarpong D. Laboratory, reading center, and coordinating center data management methods in the Jackson Heart Study. *Am J Med Sci*. 2004;328:131-44.

14. Vandenbroucke JP, von Elm E, Altman DG, G √ ∏tzsche PC, Mulrow CD, Pocock SJ, Poole C, Schlesselman JJ and Egger M. Strengthening the Reporting of Observational Studies in Epidemiology (STROBE): explanation and elaboration. *PLoS Medicine*. 2007;4:e297-e297.

15. Little J, Higgins JP, Ioannidis JP, Moher D, Gagnon F, von Elm E, Khoury MJ, Cohen B, Davey-Smith G, Grimshaw J, Scheet P, Gwinn M, Williamson RE, Zou GY, Hutchings K, Johnson CY, Tait V, Wiens M, Golding J, van Duijn C, McLaughlin J, Paterson A, Wells G, Fortier I, Freedman M, Zecevic M, King R, Infante-Rivard C, Stewart A, Birkett N and Studies STtRoGA. STrengthening the REporting of Genetic Association Studies (STREGA): an extension of the STROBE statement. *PLoS Med*. 2009;6:e22.

16. Boef AG, Dekkers OM and le Cessie S. Mendelian randomization studies: a review of the approaches used and the quality of reporting. *Int J Epidemiol*. 2015;44:496-511.

17. Williams TN, Mwangi TW, Wambua S, Alexander ND, Kortok M, Snow RW and Marsh K. Sickle cell trait and the risk of Plasmodium falciparum malaria and other childhood diseases. *J Infect Dis*. 2005;192:178-86.

18. Abdalla M, Caughey MC, Tanner RM, Booth JN, 3rd, Diaz KM, Anstey DE, Sims M, Ravenell J, Muntner P, Viera AJ and Shimbo D. Associations of Blood Pressure Dipping Patterns With Left Ventricular Mass and Left Ventricular Hypertrophy in Blacks: The Jackson Heart Study. *J Am Heart Assoc*. 2017;6:e004847-e004847.

19. Naik RP, Derebail VK, Grams ME, Franceschini N, Auer PL, Peloso GM, Young BA, Lettre G, Peralta CA, Katz R, Hyacinth HI, Quarells RC, Grove ML, Bick AG, Fontanillas P, Rich SS, Smith JD, Boerwinkle E, Rosamond WD, Ito K, Lanzkron S, Coresh J, Correa A, Sarto GE, Key NS, Jacobs DR, Kathiresan S, Bibbins-Domingo K, Kshirsagar AV, Wilson JG and Reiner AP. Association of sickle cell trait with chronic kidney disease and albuminuria in African Americans. *JAMA*. 2014;312:2115-25.

20. Austin PC and Steyerberg EW. The number of subjects per variable required in linear regression analyses. *J Clin Epidemiol*. 2015;68:627-36.

21. Thijs L, Hansen TW, Kikuya M, Bjorklund-Bodegard K, Li Y, Dolan E, Tikhonoff V, Seidlerova J, Kuznetsova T, Stolarz K, Bianchi M, Richart T, Casiglia E, Malyutina S, Filipovsky J, Kawecka-Jaszcz K, Nikitin Y, Ohkubo T, Sandoya E, Wang J, Torp-Pedersen C, Lind L, Ibsen H, Imai Y, Staessen JA, O'Brien E and Investigators I. The International Database of Ambulatory Blood Pressure in relation to Cardiovascular Outcome (IDACO): protocol and research perspectives. *Blood Press Monit*. 2007;12:255-62.

22. O'Brien E, Parati G, Stergiou G, Asmar R, Beilin L, Bilo G, Clement D, de la Sierra A, de Leeuw P, Dolan E, Fagard R, Graves J, Head GA, Imai Y, Kario K, Lurbe E, Mallion JM, Mancia G, Mengden T, Myers M, Ogedegbe G, Ohkubo T, Omboni S, Palatini P, Redon J, Ruilope LM, Shennan A, Staessen JA, vanMontfrans G, Verdecchia P, Waeber B, Wang J, Zanchetti A, Zhang Y and European Society of Hypertension Working Group on Blood Pressure M. European Society of Hypertension position paper on ambulatory blood pressure monitoring. *J Hypertens*. 2013;31:1731-68.

23. Lurbe E, Agabiti-Rosei E, Cruickshank JK, Dominiczak A, Erdine S, Hirth A, Invitti C, Litwin M, Mancia G, Pall D, Rascher W, Redon J, Schaefer F, Seeman T, Sinha M, Stabouli S, Webb NJ, Wuhl E and Zanchetti A. 2016 European Society of Hypertension guidelines for the management of high blood pressure in children and adolescents. *J Hypertens*. 2016;34:1887-920.

24. Cook RJ and Farewell VT. Multiplicity Considerations in the Design and Analysis of Clinical Trials. *JR Statist Soc A*. 1996;159:93.

25. Etyang AO, Smeeth L, Cruickshank JK and Scott JA. The Malaria-High Blood Pressure Hypothesis. *Circ Res*. 2016;119:36-40.

26. Davey Smith G and Ebrahim S. ‘Mendelian randomization’: can genetic epidemiology contribute to understanding environmental determinants of disease?*. *Int J Epidemiol*. 2003;32:1-22.

27. Stamler J, Stamler R, Rhomberg P, Dyer A, Berkson DM, Reedus W and Wannamaker J. Multivariate analysis of the relationship of six variables to blood pressure: findings from Chicago community surveys, 1965--1971. *J Chronic Dis*. 1975;28:499-525.

28. Palmer TM, Nordestgaard BG, Benn M, Tybjaerg-Hansen A, Davey Smith G, Lawlor DA and Timpson NJ. Association of plasma uric acid with ischaemic heart disease and blood pressure: mendelian randomisation analysis of two large cohorts. *BMJ*. 2013;347:f4262.

29. International Consortium for Blood Pressure Genome-Wide Association S, Ehret GB, Munroe PB, Rice KM, Bochud M, Johnson AD, Chasman DI, Smith AV, Tobin MD, Verwoert GC, Hwang SJ, Pihur V, Vollenweider P, O'Reilly PF, Amin N, Bragg-Gresham JL, Teumer A, Glazer NL, Launer L, Zhao JH, Aulchenko Y, Heath S, Sober S, Parsa A, Luan J, Arora P, Dehghan A, Zhang F, Lucas G, Hicks AA, Jackson AU, Peden JF, Tanaka T, Wild SH, Rudan I, Igl W, Milaneschi Y, Parker AN, Fava C, Chambers JC, Fox ER, Kumari M, Go MJ, van der Harst P, Kao WH, Sjogren M, Vinay DG, Alexander M, Tabara Y, Shaw-Hawkins S, Whincup PH, Liu Y, Shi G, Kuusisto J, Tayo B, Seielstad M, Sim X, Nguyen KD, Lehtimaki T, Matullo G, Wu Y, Gaunt TR, Onland-Moret NC, Cooper MN, Platou CG, Org E, Hardy R, Dahgam S, Palmen J, Vitart V, Braund PS, Kuznetsova T, Uiterwaal CS, Adeyemo A, Palmas W, Campbell H, Ludwig B, Tomaszewski M, Tzoulaki I, Palmer ND, consortium CA, Consortium CK, KidneyGen C, EchoGen c, consortium C-H, Aspelund T, Garcia M, Chang YP, O'Connell JR, Steinle NI, Grobbee DE, Arking DE, Kardia SL, Morrison AC, Hernandez D, Najjar S, McArdle WL, Hadley D, Brown MJ, Connell JM, Hingorani AD, Day IN, Lawlor DA, Beilby JP, Lawrence RW, Clarke R, Hopewell JC, Ongen H, Dreisbach AW, Li Y, Young JH, Bis JC, Kahonen M, Viikari J, Adair LS, Lee NR, Chen MH, Olden M, Pattaro C, Bolton JA, Kottgen A, Bergmann S, Mooser V, Chaturvedi N, Frayling TM, Islam M, Jafar TH, Erdmann J, Kulkarni SR, Bornstein SR, Grassler J, Groop L, Voight BF, Kettunen J, Howard P, Taylor A, Guarrera S, Ricceri F, Emilsson V, Plump A, Barroso I, Khaw KT, Weder AB, Hunt SC, Sun YV, Bergman RN, Collins FS, Bonnycastle LL, Scott LJ, Stringham HM, Peltonen L, Perola M, Vartiainen E, Brand SM, Staessen JA, Wang TJ, Burton PR, Soler Artigas M, Dong Y, Snieder H, Wang X, Zhu H, Lohman KK, Rudock ME, Heckbert SR, Smith NL, Wiggins KL, Doumatey A, Shriner D, Veldre G, Viigimaa M, Kinra S, Prabhakaran D, Tripathy V, Langefeld CD, Rosengren A, Thelle DS, Corsi AM, Singleton A, Forrester T, Hilton G, McKenzie CA, Salako T, Iwai N, Kita Y, Ogihara T, Ohkubo T, Okamura T, Ueshima H, Umemura S, Eyheramendy S, Meitinger T, Wichmann HE, Cho YS, Kim HL, Lee JY, Scott J, Sehmi JS, Zhang W, Hedblad B, Nilsson P, Smith GD, Wong A, Narisu N, Stancakova A, Raffel LJ, Yao J, Kathiresan S, O'Donnell CJ, Schwartz SM, Ikram MA, Longstreth WT, Jr., Mosley TH, Seshadri S, Shrine NR, Wain LV, Morken MA, Swift AJ, Laitinen J, Prokopenko I, Zitting P, Cooper JA, Humphries SE, Danesh J, Rasheed A, Goel A, Hamsten A, Watkins H, Bakker SJ, van Gilst WH, Janipalli CS, Mani KR, Yajnik CS, Hofman A, Mattace-Raso FU, Oostra BA, Demirkan A, Isaacs A, Rivadeneira F, Lakatta EG, Orru M, Scuteri A, Ala-Korpela M, Kangas AJ, Lyytikainen LP, Soininen P, Tukiainen T, Wurtz P, Ong RT, Dorr M, Kroemer HK, Volker U, Volzke H, Galan P, Hercberg S, Lathrop M, Zelenika D, Deloukas P, Mangino M, Spector TD, Zhai G, Meschia JF, Nalls MA, Sharma P, Terzic J, Kumar MV, Denniff M, Zukowska-Szczechowska E, Wagenknecht LE, Fowkes FG, Charchar FJ, Schwarz PE, Hayward C, Guo X, Rotimi C, Bots ML, Brand E, Samani NJ, Polasek O, Talmud PJ, Nyberg F, Kuh D, Laan M, Hveem K, Palmer LJ, van der Schouw YT, Casas JP, Mohlke KL, Vineis P, Raitakari O, Ganesh SK, Wong TY, Tai ES, Cooper RS, Laakso M, Rao DC, Harris TB, Morris RW, Dominiczak AF, Kivimaki M, Marmot MG, Miki T, Saleheen D, Chandak GR, Coresh J, Navis G, Salomaa V, Han BG, Zhu X, Kooner JS, Melander O, Ridker PM, Bandinelli S, Gyllensten UB, Wright AF, Wilson JF, Ferrucci L, Farrall M, Tuomilehto J, Pramstaller PP, Elosua R, Soranzo N, Sijbrands EJ, Altshuler D, Loos RJ, Shuldiner AR, Gieger C, Meneton P, Uitterlinden AG, Wareham NJ, Gudnason V, Rotter JI, Rettig R, Uda M, Strachan DP, Witteman JC, Hartikainen AL, Beckmann JS, Boerwinkle E, Vasan RS, Boehnke M, Larson MG, Jarvelin MR, Psaty BM, Abecasis GR, Chakravarti A, Elliott P, van Duijn CM, Newton-Cheh C, Levy D, Caulfield MJ and Johnson T. Genetic variants in novel pathways influence blood pressure and cardiovascular disease risk. *Nature*. 2011;478:103-9.

30. Cohen JC, Boerwinkle E, Mosley TH, Jr. and Hobbs HH. Sequence variations in PCSK9, low LDL, and protection against coronary heart disease. *N Engl J Med*. 2006;354:1264-72.

31. Ference BA, Robinson JG, Brook RD, Catapano AL, Chapman MJ, Neff DR, Voros S, Giugliano RP, Davey Smith G, Fazio S and Sabatine MS. Variation in PCSK9 and HMGCR and Risk of Cardiovascular Disease and Diabetes. *N Engl J Med*. 2016;375:2144-2153.

32. Khan S, Floris M, Pani A and Rosner MH. Sodium and Volume Disorders in Advanced Chronic Kidney Disease. *Adv Chronic Kidney Dis*. 2016;23:240-6.

33. de Borst MH, Vervloet MG, ter Wee PM and Navis G. Cross talk between the renin-angiotensin-aldosterone system and vitamin D-FGF-23-klotho in chronic kidney disease. *J Am Soc Nephrol*. 2011;22:1603-9.

34. Neumann J, Ligtenberg G, Klein, II, Koomans HA and Blankestijn PJ. Sympathetic hyperactivity in chronic kidney disease: pathogenesis, clinical relevance, and treatment. *Kidney Int*. 2004;65:1568-76.

35. Raine AE, Bedford L, Simpson AW, Ashley CC, Brown R, Woodhead JS and Ledingham JG. Hyperparathyroidism, platelet intracellular free calcium and hypertension in chronic renal failure. *Kidney Int*. 1993;43:700-5.

36. Passauer J, Pistrosch F, Bussemaker E, Lassig G, Herbrig K and Gross P. Reduced agonist-induced endothelium-dependent vasodilation in uremia is attributable to an impairment of vascular nitric oxide. *J Am Soc Nephrol*. 2005;16:959-65.

37. Portaluppi F, Montanari L, Massari M, Di Chiara V and Capanna M. Loss of nocturnal decline of blood pressure in hypertension due to chronic renal failure. *Am J Hypertens*. 1991;4:20-6.

38. Webster AC, Nagler EV, Morton RL and Masson P. Chronic Kidney Disease. *Lancet*. 2017;389:1238-1252.

39. Lewis JB. Blood pressure control in chronic kidney disease: is less really more? *J Am Soc Nephrol*. 2010;21:1086-92.

40. Marsh K, Forster D, Waruiru C, Mwangi I, Winstanley M, Marsh V, Newton C, Winstanley P, Warn P, Peshu N and et al. Indicators of life-threatening malaria in African children. *N Engl J Med*. 1995;332:1399-404.

41. Haycock PC, Burgess S, Wade KH, Bowden J, Relton C and Davey Smith G. Best (but oft-forgotten) practices: the design, analysis, and interpretation of Mendelian randomization studies. *Am J Clin Nutr*. 2016;103:965-78.

42. Williams TN, Mwangi TW, Wambua S, Peto TEa, Weatherall DJ, Gupta S, Recker M, Penman BS, Uyoga S, Macharia A, Mwacharo JK, Snow RW and Marsh K. Negative epistasis between the malaria-protective effects of alpha+-thalassemia and the sickle cell trait. *Nat Genet*. 2005;37:1253-7.

43. O'Meara WP, Bejon P, Mwangi TW, Okiro EA, Peshu N, Snow RW, Newton CR and Marsh K. Effect of a fall in malaria transmission on morbidity and mortality in Kilifi, Kenya. *Lancet*. 2008;372:1555-62.
